# Stability of silicon–tin alloyed nanocrystals with high tin concentration synthesized by femtosecond laser plasma in liquid media

**DOI:** 10.1038/s41598-023-33808-6

**Published:** 2023-05-17

**Authors:** Mickaël Lozac’h, Marius Bürkle, Calum McDonald, Tetsuhiko Miyadera, Tomoyuki Koganezawa, Davide Mariotti, Vladimir Švrček

**Affiliations:** 1 National Institute of Advanced Industrial Science and Technology (AIST), Central 2, Umezono 1-1-1, Tsukuba, 305-8568 Japan; 2 Japan Synchrotron Radiation Research Institute (JASRI), 1-1-1 Kouto, Sayo-cho, Sayo-gun, Hyogo 679-5198 Japan; 3 Nanotechnology and Integrated Bio-Engineering Centre (NIBEC), University of Ulster, Belfast, UK

**Keywords:** Electronic properties and materials, Nanoparticles, Structural properties, Synthesis and processing

## Abstract

Nanocrystals have a great potential for future materials with tunable bandgap, due to their optical properties that are related with the material used, their sizes and their surface termination. Here, we concentrate on the silicon–tin alloy for photovoltaic applications due to their bandgap, lower than bulk Si, and also the possibility to activate direct band to band transition for high tin concentration. We synthesized silicon–tin alloy nanocrystals (SiSn-NCs) with diameter of about 2–3 nm by confined plasma technique employing a femtosecond laser irradiation on amorphous silicon–tin substrate submerged in liquid media. The tin concentration is estimated to be $\sim 17\%$, being the highest Sn concentration for SiSn-NCs reported so far. Our SiSn-NCs have a well-defined zinc-blend structure and, contrary to pure tin NCs, also an excellent thermal stability comparable to highly stable silicon NCs. We demonstrate by means of high resolution synchrotron XRD analysis (SPring 8) that the SiSn-NCs remain stable from room temperature up to $400\,^{\circ }\text {C},$ with a relatively small expansion of the crystal lattice. The high thermal stability observed experimentally is rationalized by means of first-principle calculations.

## Introduction

Since the first modern photovoltaic devices have been introduced in the 1950s^1^ their efficiency has largely improved from 6% to up to 27% for state-of-the-art silicon photovoltaic devices^2^. The recent improvement, however, can mainly be attributed to improved processing techniques and clever engineering as we are reaching the intrinsic limitation of silicon-based photovoltaics. Namely, the indirect and difficult to tune bandgap of silicon and the limited multi-exciton generation (MEG) in bulk materials^3,4^.

Nanocrystals with quantum confinement could offer a solution as their optical properties can be readily tuned by the interplay between the semiconductor material, the size of NCs, and their surface properties^5–9^. Nanocrystals could thus overcome both aforementioned problems, as we have a large degree of control over the bandgap properties^4,8,10–12^ and strong quantum confinement in small NCs that can largely improve MEG due to enhanced inverse Auger recombination^3,13–15^.

Silicon–tin alloyed nanocrystals (SiSn-NCs) are of particular interest because they remain compatible with the existing silicon technology. Alloying with the group IV element Sn^16,17^ is not only promising for reducing the bandgap but also to modify its nature from indirect to direct^18^. Tin and specifically $\alpha$-Sn^19,20^ has a much smaller bandgap than silicon^20^ with the same crystal structure, i.e. a diamond cubic structure, which largely avoids problems such as lattice mismatch, dislocations, and defects at interfaces^21,22^.

**Figure 1:**
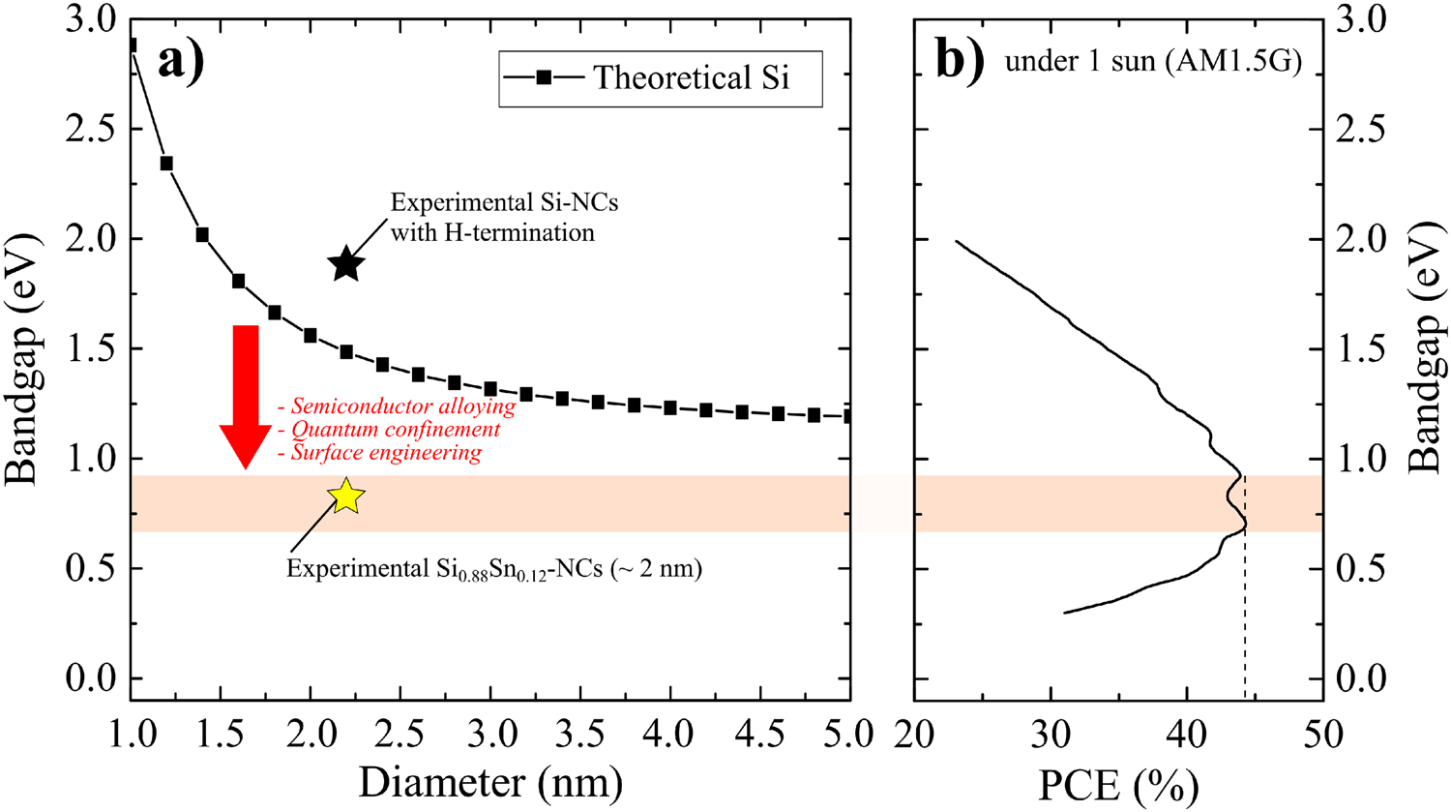
(**a**) Calculation of the Si-NCs bandgap (squares) as a function of NCs size by using the Brus equation^10^. The experimental bandgap of Si-NCs and SiSn-NCs with Sn concentration of $12\%$ are also presented (stars)^9^. (**b**) The maximum photo-conversion efficiency (PCE) for solar cells under one sun (AM 1.5G spectra) using multiple exciton generation (MEG) is presented as a function of the semiconductor bandgap^23^, where the maximum PCE of $44\%$ is underlined by the colored bar for a bandgap range from $0.7\,\text {eV}$ to $0.9\,\text {eV}$.

Figure 1 illustrates the purpose of SiSn-NCs in photovoltaic devices. The Brus equation^10^ (see Supporting Information) shows that reducing the diameter of Si-NCs increases the bandgap (Fig. 1a) but moves it away from maximum photoconversion efficiency achievable by MEG (Fig. 1b)^23^. SiSn-NCs combine quantum confinement effects with semiconductor alloying and surface engineering to address this issue^8^, resulting in an optical bandgap of $0.81\,\text {eV}$ for 2 nm diameter Si$_{{0.88}}$ Sn$_{{0.12}}$-NCs^9^. However, Si$_{{0.88}}$Sn$_{{0.12}}$-NCs still most likely present an indirect transition^18^, requiring new fabrication processes allowing for higher tin concentration. The impact of the quantum confinement on the bandgap of SiSn-NCs, supported by the density functional theory with an approximate (quasi) band structure for finite-sized nanocrystals, underlines a transition from indirect to direct bandgap for SiSn-NCs only for high tin concentration above 41%^18^.

However, the fabrication of Sn-alloyed Si nanocrystals remains a technological challenge^24^ due to the difference in atomic size between Si and Sn (covalent radius are 111 p.m. for Si and 139 p.m. for Sn^25^) and the presence of metallic phase of tin ($\beta$-Sn)^20^. Besides, the investigation of the thermal stability of SiSn-NCs is also a crucial step prior to their integration in solar cell fabrication. $\beta$-Sn has a relatively low melting point of about $234\,^{\circ }\text {C}$^26^ which is well below the temperature ($\sim 300\,^{\circ }\text {C}$) required for existing silicon processing technologies^27,28^. We demonstrated that highly non-equilibrium and spatially confined short pulsed laser process in liquid media^29^ can be used to fabricate SiSn alloyed NCs with sizes in the strong quantum confinement regime^6^ and we were able to embed them into a polymer layer in order to characterize their photovoltaic properties^9^.

This study attempts to overcome the challenging fabrication process of SiSn-NCs by utilizing a confined plasma generated from femtosecond (fs) laser in liquid media, as opposed to prior studies that employed nanosecond (ns) laser plasma^9^. Additionally, this work aims to evaluate the thermal stability of the fabricated SiSn-NCs, as this is a critical factor for their potential incorporation in next-generation photovoltaic devices. We successfully obtained SiSn-NCs with a high tin concentration of $17\%$ by confined plasma from fs laser, which is, to the best of our knowledge, the highest Sn concentration in crystalline form. To show that the SiSn-NCs are stable at the required processing temperatures ($\sim 300\,^{\circ }\text {C}$), we determined the melting point of the alloyed SiSn-NCs besides the ones of the pure Si-NCs and the $\beta$-Sn-NCs. Using high resolution (HR-) XRD with $1\,{\text{\AA }}$ synchrotron radiation (SPring-8), which allows a precise analysis of the NCs regardless of the presence of other crystallites^9^, we find that SiSn-NCs remains stable up to $400\,^{\circ }\text {C}$, while $\beta$-Sn-NCs already decompose at about $200\,^{\circ }\text {C}$. Lastly, to rationalize the thermal stability of SiSn-NCs observed experimentally, we correlate their stability with the cohesive and formation energies necessary for their fabrication using first-principle calculations^8,18^. These findings demonstrate that the highly thermally stable SiSn-NCs can indeed be compatible with existing silicon processing technologies.

## Experimental results and discussion

**Figure 2:**
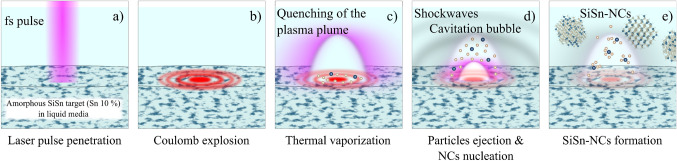
Illustration of the formation of SiSn-NCs in 5 steps from (**a**) to (**e**) during femtosecond (fs) laser processing on amorphous SiSn target in liquid media (DI water). The amorphous target has a tin concentration of 10%.

Here we use a Femtosecond (fs) laser generated plasma to fabricate SiSn-NCs in liquid media. The interested reader is referred to references^30–32^ for a detailed discussion. The basic idea is that a fs pulsed laser generates a cold (non-thermal) plasma that prevents migration of dopants to the NC surface^32,33^ allowing for much higher dopant concentrations than traditional methods. Figure 2 sketches the fabrication process. The fs laser pulse is absorbed (multiphoton absorption) by the SiSn target (Fig. 2a). The resulting Coulomb explosion (Fig. 2b) creates a small plasma cloud with energetic ions with higher velocities than in thermal emission (e.g. ms/ns pulsed lasers). The plasma is confined inside a cavitation bubble with Si and Sn atoms ejected due to the shockwaves propagation inside the plasma and the material (Fig. 2d). The ejected Si and Sn are quickly thermalized due to the surrounding liquid and start to nucleate into nanocrystals, and NCs grow to form SiSn-NCs (Fig. 2e). In this step, due to the very fast plume condensation and the high pressure (in the order of GPa) loss of dopants to the surrounding environment is largely suppressed^32–34^ . The fs-laser used in this work has a peak pulse power of 0.84 GW, about 2 orders of magnitude higher than the ns-laser used in previous studies.

**Figure 3:**
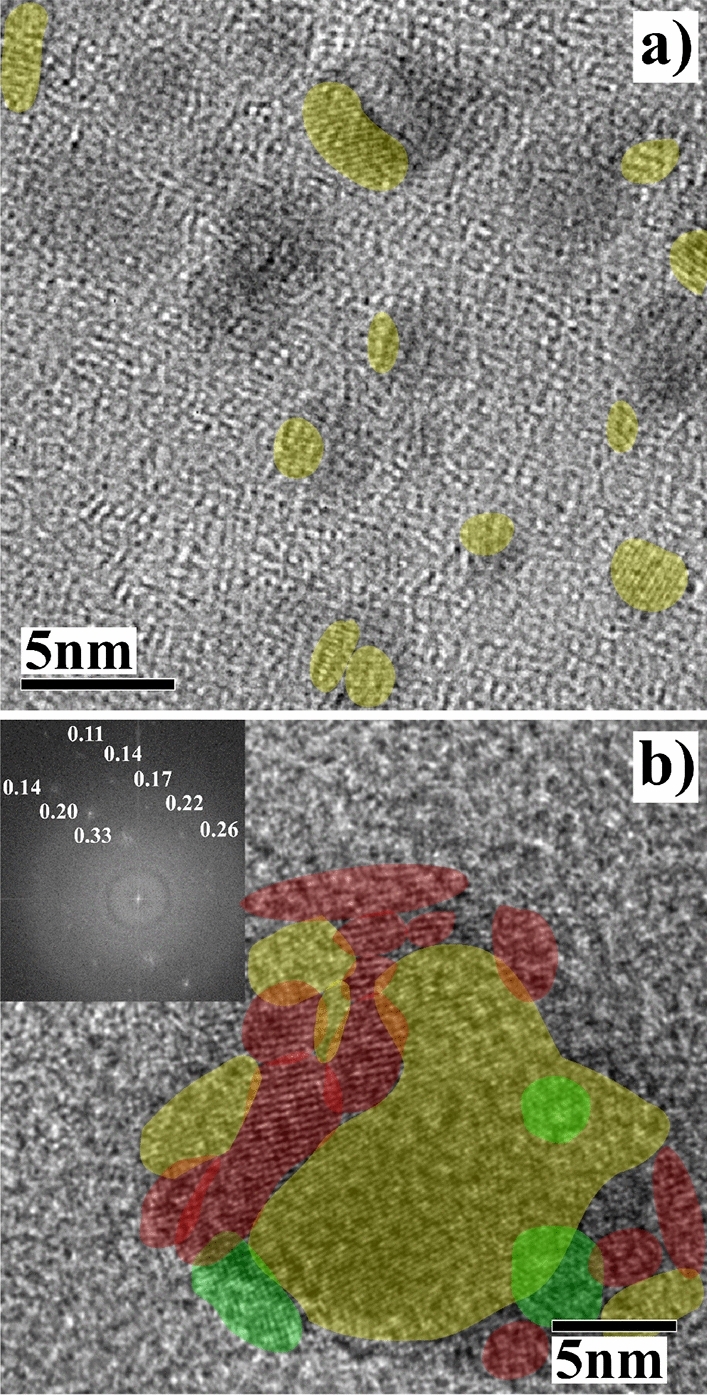
(**a**) TEM image of SiSn-NCs about 2–3 nm fabricated by femtosecond laser plasma in liquid media. (**b**) Cluster of larger SiSn-NCs, with different colors representing different atomic plans, used for fast Fourier transform (FFT) image analysis, the inset presents the FFT image with the distances displayed in nm between various atomic plans (d-spacing).

**Figure 4:**
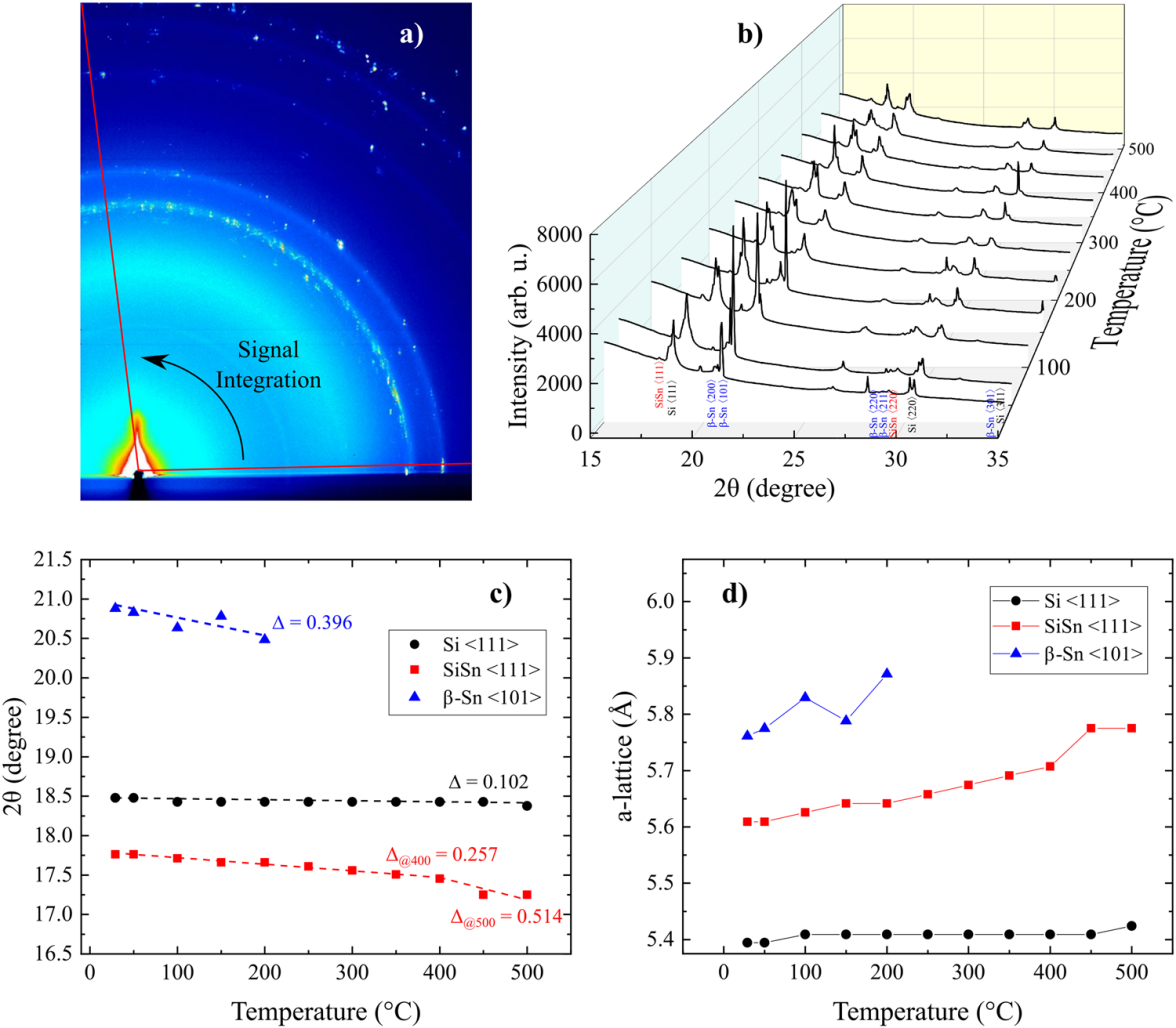
High resolution (HR-)XRD analysis, using synchrotron radiation at SPring 8 (wavelength of $1\,{\text{\AA }}$), of SiSn-NCs fabricated by fs-laser plasma as a function of the annealing temperature from 29 to $500\,^{\circ }\text {C}$. (**a**) Raw image of diffracted X-rays from SiSn-NCs annealed at $50\,^{\circ }\text {C}$. (**b**) Corresponding HR-XRD spectra with a signal integrated from $2^{\circ }$ to $98^{\circ }$ of SiSn-NCs annealed from 29 to $500\,^{\circ }\text {C}$. (**c**) The peaks positions of Si $\langle 111\rangle$, SiSn $\langle 111\rangle$, and $\beta$-Sn $\langle 101\rangle$ are reported as a function of the temperature to underline the shift observed for the main peaks, and (**d**) the corresponding a-lattices.

The drawbacks of this fabrication process are the relatively large size distribution of the NCs obtained, and the presence of other crystallites^9^. Of course such a process will require optimization to maximize the yield of these crystallites, which is outside the scope of this work. For example, by using ns-laser plasma where most of the Si$_{0.88}$Sn$_{0.12}$-NCs have a diameter about 2–3 nm, the size distribution is estimated to be 1–8 nm^9^. Here, we focus on SiSn-NCs with a diameter between 2 and 3 nm generated by fs laser plasma. Figure 3a presents the TEM image of SiSn-NCs fabricated by fs laser plasma, the aligned atomic planes are highlighted and represent SiSn-NCs with a size of about 2–3 nm. However for such a small crystal size, there are too few atomic planes to analyze the NCs by Fast Fourier Transform (FFT). Figure 3b represents a cluster of SiSn-NCs with a large diameter of about 25 nm used for FFT measurements. The inset of Fig. 3 shows the FFT image with the distances displayed in nm between various atomic planes (d-spacing). The average *a*-lattice constant is estimated by FFT to be about $5.66\pm 0.07\,{{\text{\AA }}}$ (method provided in Supporting Information), then the Sn concentration is determined using Vegard’s law^35^ from Eq. 1^36^ without the bowing parameter (b = 0), assuming a linear interpolation

1 $$\begin{aligned} a_{Si_{1-x}Sn_{x}}=xa_{\alpha -Sn}+(1-x)a_{Si}-bx(1-x) \end{aligned}$$

where *x* is the Sn concentration, and $a_{Si_{1-x}Sn_{x}}$, $a_{\alpha -Sn}$, and $a_{Si}$ are the a-lattice constants of the alloyed SiSn, $\alpha$-Sn, and silicon, respectively. The Sn concentration was estimated to be $21\pm 6\%$, using the following lattice constants $a_{Si}=5.43071\,{\text{\AA }}$^37,38^ and $a_{\alpha -Sn}=6.4912\,{\text{\AA }}$^20^. The discrepancy of the average a-lattice by FFT gives a large uncertainty for the Sn concentration. Moreover, the Sn concentration corresponds to a specific cluster that may not represent most of the NCs fabricated. Thus, we performed HR-XRD using synchrotron radiation with a wavelength of $1\,{\text{\AA }}$ at SPring 8 (see the “Method” section) to evaluate the Sn concentration of a large quantity of NCs. The very short wavelength of $1\,{\text{\AA }}$ allows us to estimate precisely the d-spacing and thus the a-lattice constant, which is not possible using conventional XPS analysis^39^. The amount of NCs analyzed in a single measurement is in the order of $10^{15}$ NCs (see the “Method” section). The peak position, at room temperature, of SiSn $\langle {111}\rangle$ is measured at $17.76\pm 0.01^{\circ }$ (Fig. 4), which corresponds to an a-lattice constant of $5.610\pm 0.003\,{\text{\AA }}$ and a Sn concentration estimated to be $16.9\pm 0.3\%$ (method provided in Supporting Information). Thus, by using the femtosecond laser process, the Sn concentration of SiSn-NCs alloy was enhanced up to $17\%$, which is, to the best of the our knowledge, the highest Sn concentration reported for SiSn alloyed NCs so far.

To analyze the thermal stability of SiSn-NCs fabricated by fs-laser plasma, we perform HR-XRD analysis to determine in situ the lattice parameters during annealing at different temperatures (Fig. 4). The same sample contains an amount of $10^{15}\,\text {NCs}$ that is heated from 29 to $500\,^{\circ }\text {C}$. Figure 4a shows a raw image of the diffracted X-rays from the SiSn-NCs annealed at $50\,^{\circ }\text {C}$. Each spectrum is the integrated signal with an angle of $96^{\circ }$ from an XRD image at a given temperature (see the full set of HR-XRD images in Supporting Information). In Fig. 4b the corresponding 2$\theta$ scan spectra from 29 to $500\,^{\circ }\text {C}$ are summarized. The atomic planes from the following structures are well defined and can readily be identified: pristine Si, metallic $\beta$-Sn, and SiSn alloy. The 2$\theta$ peak positions of the main peaks Si $\langle 111\rangle$, SiSn $\langle 111\rangle$, and $\beta$-Sn $\langle 101\rangle$ are reported as a function of the annealing temperature in Fig. 4c. The atomic planes $\langle 111\rangle$, and $\langle 220\rangle$ of the pristine Si are essentially not perturbed by temperature and remain at the same position for the full temperature range studied, underlined by the peak position of Si $\langle 111\rangle$ in Fig. 4c with a small variation between the peak positions 2$\theta$ at $29\,^{\circ }\text {C}$ and $500\,^{\circ }\text {C}$ ($\Delta \approx 0.1^{\circ }$, (Fig. 4c) translating to a slight expansion in terms of a-lattice constant ($\Delta a<0.03\,{\text{\AA }}$, Fig. 4d). This is not the case for the metallic $\beta$-Sn where the atomic planes $\langle 200\rangle$, $\langle 101\rangle$, and $\langle 220\rangle$ are shifted to lower 2$\theta$ values with increasing temperature. The peak position of $\beta$-Sn $\langle 101\rangle$ exhibits a variation of $\Delta \approx 0.4^{\circ }$ (Fig. 4c) representing a significant expansion of the a-lattice constant $\Delta a=0.11\,{\text{\AA }}$ (Fig. 4d). Furthermore the high intensity $\beta$-Sn$\langle 101\rangle$ peak quickly disappears for temperatures above $200\,^{\circ }\text {C}$ (Fig. 4b), which is reasonable considering that $\beta$-Sn melts at $231.93\,^{\circ }\text {C}$^40^. Interestingly, the SiSn atomic plan $\langle 111\rangle$ is stable with a constant shift observed up to $400\,^{\circ }\text {C}$ ($\Delta \approx 0.26^{\circ }$, $\Delta a=0.10\,{\text{\AA }}$), above $400\,^{\circ }\text {C}$ the shift to lower 2$\theta$ is more pronounced with $\Delta =0.51^{\circ }$ ($\Delta a=0.17\,{\text{\AA }}$) (Fig. 4c,d). Overall, the alloyed Si$_{{0.83}}$Sn$_{{0.17}}$-NCs fabricated by fs-laser plasma show an excellent thermal stability up to $400\,^{\circ }\text {C}$, which is well above the thermal stability of $\alpha$-Sn, not stable at room temperature, but also $\beta$-Sn that melt above $234\,^{\circ }\text {C}$^26^. This exceptional thermal stability up to $400\,^{\circ }\text {C}$ is also well above the temperatures necessary for the fabrication processes of photovoltaic devices such as the heterojunction (HJ) solar cell structure with a hydrogenated amorphous silicon (a-Si:H) layer^41,42^. For temperatures above $400\,^{\circ }\text {C}$, the larger shift observed for SiSn-NCs may be attributed to a diffusion of Sn atoms to the surface of NCs.

An other advantage of the femtosecond laser concerns the low amount of oxidized species with a low peak intensity at $26.4^{\circ }$ corresponding to the cristobalite or tridymite form of SiO$_{{2}}$, and without SnO or SnO$_{{2}}$ contrary to the NCs using nanosecond laser^9^.

**Figure 5:**
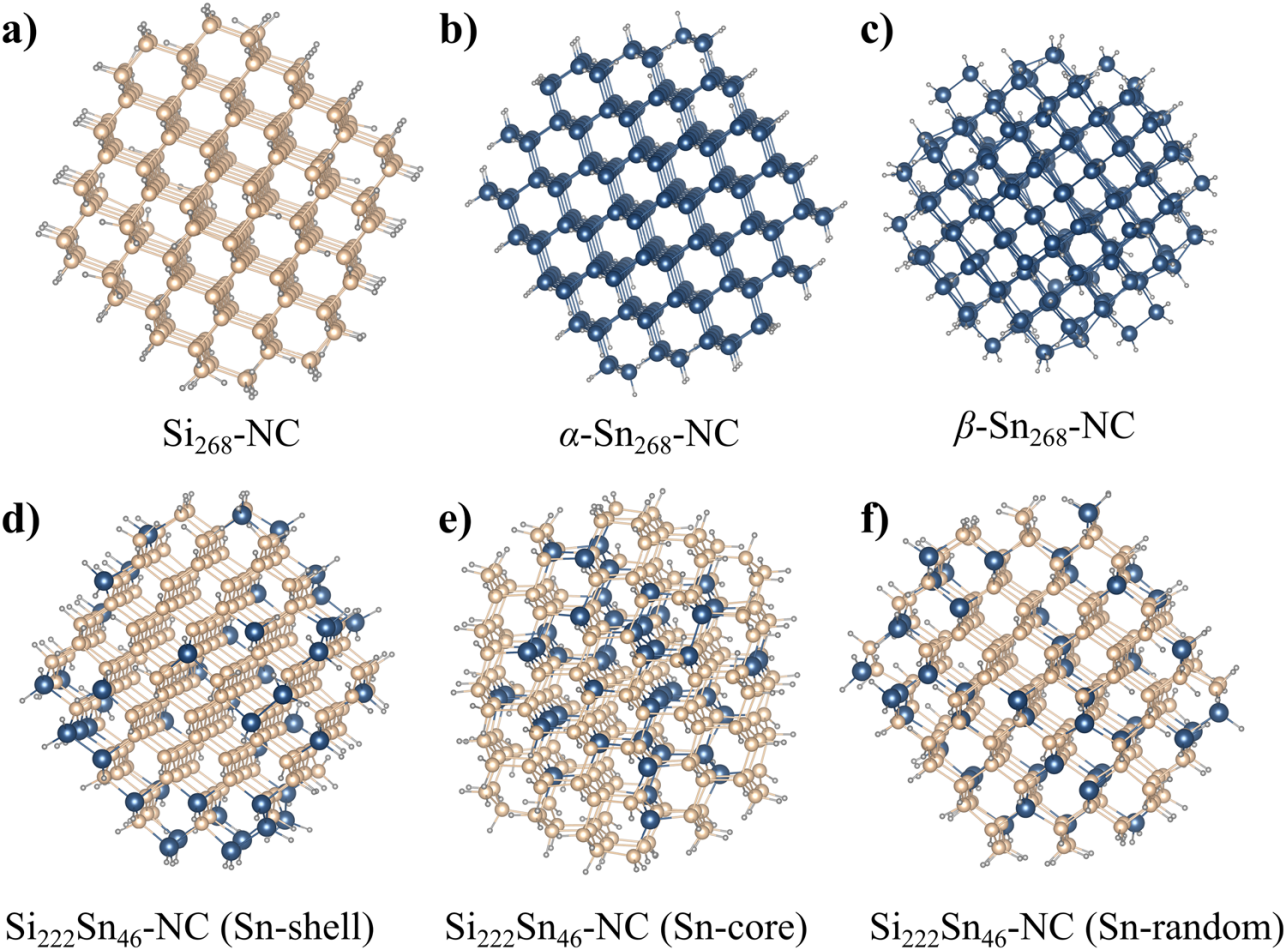
Nanocrystal geometries of the fully relaxed structures, namely (**a**) Si-NCs, (**b**) $\alpha$-Sn-NCs, and Si$_{{0.83}}$Sn$_{{0.17}}$-NCs with Sn atoms (**c**) on the NC surface, (**d**) inside the NC core, and (**e**) randomly distributed. The particle diameter is for all structures $d\approx 2.0\,\text {nm}$. Beige spheres represent Si atoms, blue spheres Sn atoms, and gray spheres H atoms.

**Table 1:** Summary of the calculated cohesive energies $E_{C}$ per atom, relative formation energies $\Delta E_{F}$ per atom, and relative energy difference of the alloyed NC with respect to the pure homoatomic NCs $E_{Mix}$per atomfor the different NCs structures illustrated in Fig. 5.

	Si$_{{268}}$	Si$_{{222}}$Sn$_{{46}}$(17%Sn-shell)	Si$_{{222}}$Sn$_{{46}}$(17% Sn-core)	Si$_{{222}}$Sn$_{{46}}$(17%Sn-random)	$\alpha$-Sn$_{{268}}$	$\beta$-Sn$_{{268}}$
$E_{C}$/atom (eV)	3.71	3.46	3.48	3.47	2.59	2.43
$\Delta E_{F}$/atom (eV)	/	0.26	0.22	0.24	/	/
$E_{Mix}$/atom (eV)	/	$-0.058$	$-0.032$	$-0.045$	/	/
Estimated bandgap (eV)	1.94	1.53	1.80	1.76	1.18	0.12

The estimated bandgap is also presented, which corresponds to the gap between the highest occupied molecular orbital (HOMO) and the lowest unoccupied molecular orbital (LUMO) of the cluster. The number of hydrogen atoms is omitted in the description.

To understand the thermal stability of SiSn-NCs observed experimentally, we calculate the cohesive and the formation energies of pristine Si-, $\alpha$-Sn-, $\beta$-Sn-, and Si$_{{83\%}}$Sn$_{{17\%}}$-NCs by means of first-principle calculations. The particle size is comparable to the experimental result of about $\sim 2\,\text {nm}$ corresponding to a $\text {Si}_{x}\text {Sn}_{y}$ NC with $x+y=268$ atoms. Moreover, we assume the surface to be fully passivated with hydrogen atoms. For the NCs with diamond cubic lattice, i.e., $\text {Si}_{268}$, $\text {Si}_{222}\text {Sn}_{46}$, and $\alpha \text {-Sn}_{268}$ this corresponds to $n=156$ H atoms and for the body-centered tetragonal $\beta \text {-Sn}_{268}$ to $n=198$ H atoms. To model the nanocrystal geometry we start from the corresponding bulk lattice and generate an initial structure with the desired radius (origin at centroid of the bulk primitive unit cell) by cutting the NC out from the ideal bulk structure. Undercoordinated surface atoms are removed to obtain well-defined crystal surfaces and the dangling bonds of the surface atoms are fully passivated by hydrogen. The geometries of these initial structures are then fully optimized, the resulting structures are summarized in Fig.  5. The computational details are given below and the Cartesian coordinates of the NCs can be found in the ESI. As it is impossible to distinguish experimentally whether NCs are formed with tin atoms preferentially substituted inside the NC core or at the NC’s surface, we will consider both cases in our calculations. The cohesive energy is defined as the energy gain by condensing the atoms from gas-phase into solid-phase^43,44^:

2 $$\begin{aligned} E_{C}&= -\bigl \{ E_{Si_{x}Sn_{y}}-xE_{Si}-yE_{Sn} \\ & -nE_{H}\bigr \}, \end{aligned}$$

with *x*, *y*, and *n* being the number of Si, Sn, and H atoms in the NC. The formation energy $E_{F}$ relative to the pristine silicon nanocrystal, is given by:

3 $$\begin{aligned} \Delta E_{F}=E_{Si_{x+y}}-E_{Si_{x}Sn_{y}}+y\left( E_{Sn}-E_{Si}\right) . \end{aligned}$$

Accordingly we can define the relative energy difference of the alloyed NC with respect to the pure homoatomic NCs^43^

4 $$\begin{aligned} E_{Mix}&= -\left\{ E_{Si_{x}Sn_{y}}-\dfrac{x}{x+y}E_{Si_{x+y}}\right. \\ & \left. -\dfrac{y}{x+y}E_{Sn_{x+y}}\right\} \end{aligned}$$

here $E_{Sn_{x+y}}$ corresponds to the energy of the $\alpha$-Sn NC which has the same diamond cubic lattice as the silicon NC.

The calculated $E_{C}$, $\Delta E_{F}$, and $E_{Mix}$ per atom are summarized in Table 1 and the corresponding optimized structures are presented in Fig. 5. While the cohesive energy of the pure Si and $\alpha \text {-Sn}$ clusters is smaller than the bulk values $E_{C}^{\text {Si-bulk}}=4.63$ and $E_{C}^{\alpha \text {-Sn-bulk}}=3.14$^45–47^, the corresponding ratio of the cohesive energies of the pure NCs $E_{C}^{\text {Si}_{268}}/E_{C}^{\alpha \text {-Sn}_{268}}=1.43$ remains close to the bulk value $E_{C}^{\text {Si-bulk}}/E_{C}^{\alpha \text {-Sn-bulk}}=1.43$. The formation of pure silicon clusters is energetically clearly preferred over the formation of pure tin clusters. Assuming substitutional Sn doping, we can roughly distinguish three distinct substitutions sites which all give comparable formation energies for the substitution of a single Si atom with Sn, namely (i) $E_{F}=2.25\,\text {eV}$ for a (100) surface site with 2 nearest neighboring Si atoms; (ii) $E_{F}=2.43\,\text {eV}$for a (111) surface site with 3 nearest neighboring Si atoms; and (iii) $E_{F}=2.10\,\text {eV}$ for inside the nanocrystal with 4 nearest neighboring Si atoms. Increasing the Sn doping to $17\%$ decreases the magnitude of the cohesive energy and accordingly raises the formation energy (Table 1), however the difference in $E_{C}$ and $E_{Mix}$ between pure Si and alloyed SiSn nanocrystals remains moderate and we can expected that both species are readily formed inside the plasma and their thermal stability can be expected to be roughly comparable as the melting temperature is proportional to the cohesive energy^48^. Accordingly, due to the smaller magnitude of the cohesive energy for pure tin NCs their melting temperature is largely reduced. The estimated bandgap of these NC structures are also reported in Table 1, it is worth noting that the values of bandgap calculated from first-principle calculations overestimated the experimental bandgaps, however it reveals a clear tendency of a reduction in the bandgap when alloying the NC. Additionally it is worth noting that, while self-purification for small NCs has been suggested^49^ it remains speculative, and is controversially discussed^50,51^. Here, due to the small energetic difference between surface and core Sn atoms, self-purification seems unlikely as the defect diffusion remains very limited especially given the very short timescales of NC formation in the fs-laser process. Thus we expect doping of Si NCs to be readily possible which is consistent with previous studies on Si NCs^52–54^.

## Conclusion

In this work, we fabricated nanocrystals of silicon–tin alloy (SiSn-NCs) by femtosecond laser plasma in liquid media as a high potential for future solar cell devices. These advanced solar cells could take advantage of multiple exciton generation (MEG) that can be triggered at a lower bandgap than the silicon bandgap. SiSn-NCs fabricated by fs-laser plasma presents a tin concentration estimated about $17\%$ by high resolution XRD with synchrotron radiation, with most of NCs having a diameter of about 2–3 nm. By using HR-XRD during thermal annealing in situ, we underlined the thermal stability of Si$_{0.83}$Sn$_{0.17}$-NCs up to $400\,^{\circ }\text {C}$. Finally the cohesive and formation energies of Si$_{{0.83}}$Sn$_{{0.17}}$-NCs for substitutional tin inside the NC core are lower than the substitutional sites on the surface and the pure $\alpha$-Sn, and thus substitutional Sn preferentially formed in the Si NC core during the fabrication process.

## Method

### SiSn-NCs fabrication

SiSn-NCs are fabricated by femtosecond laser (Libra Solo Ultrafast Optical Parametric Amplifier-OPerA) ablation in deionized (DI) water of a 5 mm thick amorphous Si$_{0.9}$Sn$_{0.1}$-NCs target. The target is immersed in 6 ml of high purity DI water with a resistivity of about$15 \textrm{M} {\Omega }$. The peak pulse power is calculated to be about 0.84 GW using a measured energy of $70\,{{\upmu \textrm{J}}}$, with a pulse of 83 fs at 1 kHz frequency, and a wavelength of 360 nm. The focal point area of the laser beam on the target is estimated to be approximately 1 mm$_{2}$. The target is rotated for better homogeneity and to avoid irradiating the exact same spot for the next pulse. The amount of nanocrystal fabricated for 1 h of irradiation by femto-second laser is about 0.5 mg. Therefore, we need several runs to create a sufficient amount of nanocrystal that were analyzed by high-resolution X-ray diffraction. It has been shown that the laser pulse is important to determine the Si–Sn relative concentration where previous work with nano-second lasers could only reach $12\%$ of Sn concentration^9^. Also varying the Sn concentration in the amorphous target could provide further opportunity for composition tuning. In order to achieve higher purity of the desired SiSn-NCs, an optimization procedure would be required, where aspects that relate to target preparation, laser process parameters and post-synthesis purification should be considered. In particular, we feel that the target plays a role, and this should be characterized and produced to facilitate a more homogeneous layer. The use of different solvent compositions and fluid-dynamics conditions should also be explored. Separation methodologies via centrifugation may also come useful. However, overall, our findings have confirmed that high Sn concentration Si-Sn phases are possible and stable.

### TEM measurements

NCs are examined by TEM and SAED using a JEOL JEM-2100F electron microscope operated at 200 keV. The samples for TEM are prepared by drop casting NCs/water colloids on holy carbon mesh/Cu TEM grids and are then allowed to dry. The analysis of the size distribution of the SiSn-NCs is carried out by processing TEM micrographs with ImageJ software. The lattice fringes are measured using Gatan Microscopy Suit Software while the analysis of SAED patterns is analyzed manually by measuring the diameters of each ring and comparing them with standard crystallographic JCPDS cards.

### XRD analysis

The high resolution X-ray diffraction measurements are performed with a synchrotron radiation at the beamline BL46XU Spring 8. The energy beam is precisely controlled at 12.938 keV corresponding to a wavelength of $1 {\text{\AA }}$. The NCs are inserted inside a quartz tube with a diameter of 0.5 mm and a thickness about 0.01 mm. The diameter of the beam was about $5\,{\mu } {\textrm{m}}$. Thus, the volume analyzed is estimated about $9.05\times 10^{-1}\,\text {mm}^{3}$, which represents up to $10^{15}$ nanocrystals with 3 nm diameter. This amount gives a good average for the determination of their structures and optical properties.

### First-principle calculations

To model the nanocrystal geometry we generate an initial structure with the desired radius (origin at centroid of the unit cell) cutout from the corresponding bulk lattice (diamond cubic lattice for Si and $\alpha$-Sn and body-centered tetragonal for $\beta$-Sn) with undercoordinated surface atoms removed, and fully passivated by hydrogen. Within density functional theory (DFT), as implemented in the first-principles package *Turbomole*^55^, the resulting structures are fully relaxed until the maximum norm of the residual force drops below $10^{-4}\,\text {a.u.}$, whereby the calculations are performed at the PBE level of theory^56,57^ using the def2-SV(P)^58^ and the respective Coulomb fitting basis^59^.

## Supplementary Information

Supplementary Information.

## Data Availability

The dataset generated and/or analyzed during the current study are available in the figshare repository: https://doi.org/10.6084/m9.figshare.21878316.v1.
