# Supplementary material for: Stability of silicon–tin alloyed nanocrystals with high tin concentration synthesized by femtosecond laser plasma in liquid media
Source: Sci Rep. 2023 May 17;13:7958. doi: 10.1038/s41598-023-33808-6 (PMC10192323; doi:10.1038/s41598-023-33808-6)
Supplement: Supplementary file 1 — Supplementary Information. [file 41598_2023_33808_MOESM1_ESM.pdf]

Supporting information - Stability of Silicon-Tin Alloyed Nanocrystals with High Tin Concentration synthesized by Femtosecond Laser Plasma in Liquid Media

Mickaël Lozac'h,[*,†] Marius Bürkle,[†] Calum McDonald,[†] Tetsuhiko Miyadera,[†] Tomoyuki Koganezawa,[‡] Davide Mariotti,[¶] and Vladimir Švrček[†]

†*National Institute of Advanced Industrial Science and Technology (AIST), Central 2, Umezono 1-1-1, Tsukuba, 305-8568, Japan*

‡*Japan Synchrotron Radiation Research Institute (JASRI), 1-1-1 Kouto, Sayo-cho, Sayo-gun, Hyogo 679-5198, Japan*

¶*Nanotechnology & Integrated Bio-Engineering Centre (NIBEC), University of Ulster, UK*

E-mail: mickael.lozach@aist.go.jp

Brus equation. The Brus equation underlines a bandgap opening when the diameter of the NCs decreases. We calculate the bandgap modulation of pristine silicon nanocrystals (Si-NCs) as a function of the diameter following the Brus equation[S1]

$$\Delta E = \frac{\hbar^2 \pi^2}{2R^2}\left[\frac{1}{m_e} + \frac{1}{m_h}\right] - \frac{1.8 e^2}{\epsilon R}, \tag{1}$$

where ΔE is the bandgap difference between the nanocrystals and the bulk semiconductor, R is the radius of nanocrystals, $m_e = 0.19\, m_0$ and $m_h = 0.49\, m_0$ are the electron and hole effective mass[S2], respectively, with m_0 the electron mass, e is the elementary charge of the

electron, and ϵ the dielectric constant obtained with $\epsilon = \epsilon_{Si}\epsilon_0$ where $\epsilon_{Si} = 11.68$ is the dielectric constant of silicon[S3] and ϵ_0 is the vacuum permittivity. For simplicity, we keep ϵ constant using ϵ_{Si} and neglecting a possible size dependence[S4].

High-resolution XRD images. The full set of HR-XRD images realized with synchrotron radiation (wavelength of 1 Å) at Spring 8 is presented in Fig. S1. The images are integrated from an angle of 2° to 98° to obtain the XRD spectra using the software Pilatus at Spring 8. Another visualization of the 2θ spectra underlining the main peak positions is shown in Fig. S2. The black arrows underline the shift to lower 2θ peak positions with the annealing temperature observed for β-Sn $\langle 200 \rangle$, $\langle 101 \rangle$, $\langle 220 \rangle$, and $\langle 211 \rangle$.

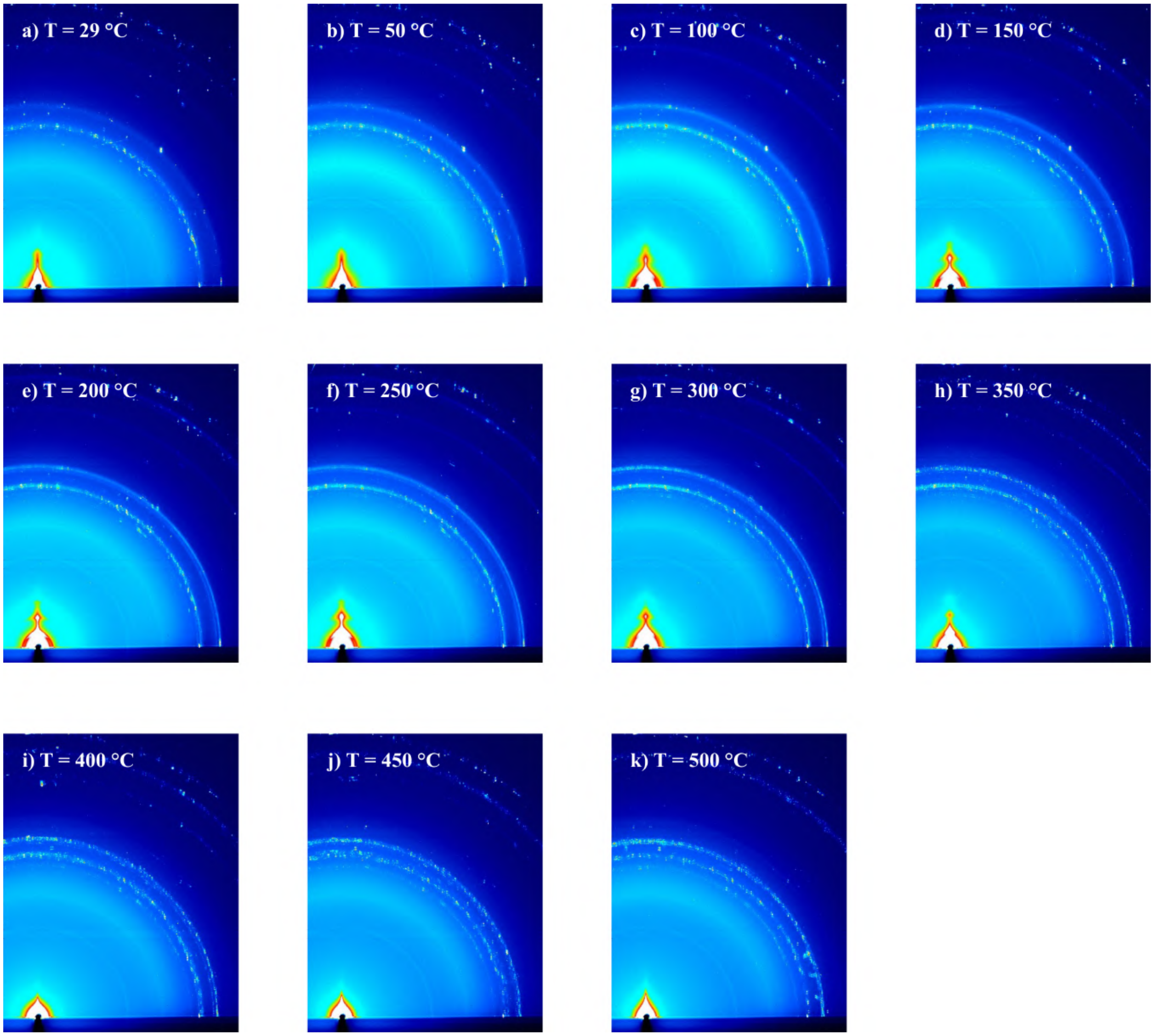

Figure S1: Full set of High resolution (HR-)XRD spectra, using synchrotron radiation at SPring 8 with a wavelength of 1 Å. Figures SS1a to SS1k present the image of diffracted XRD from NCs as a function of the annealing temperature from 29 °C to 500 °C.

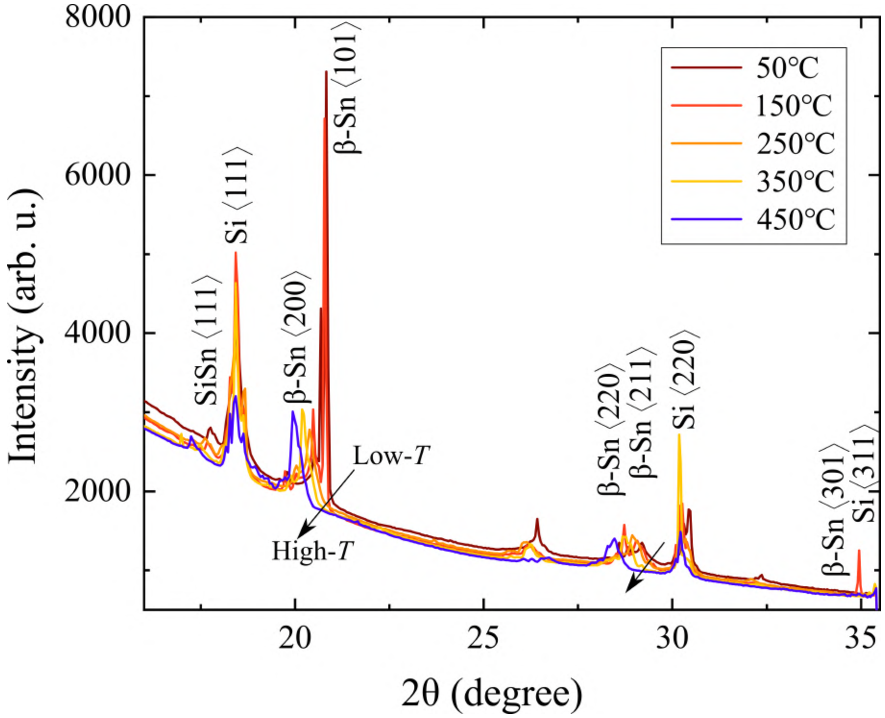

Figure S2: High resolution (HR-)XRD spectra, using synchrotron radiation at SPring 8 with a wavelength of 1 Å, of SiSn-NCs fabricated by fs-laser plasma in function of the annealing temperature. For clarity, only the spectra at 50 °C, 150 °C, 250 °C, 350 °C, and 450 °C are reported.

Calculation of Sn concentration from HR-XRD peak position. Considering the 2θ peak measured at 17.763 °, we need to determine the corresponding d-spacing, i.e. the distance between atomic planes. From the equation of the Bragg diffraction[S5]:

$$d = \frac{n\lambda}{2\sin(\theta)}, \tag{2}$$

where n is an integer set at 1, λ is the wavelength of the XRD set at 1 Å, and θ is the measured position of the peak considered. The d-spacing (d in 2) is calculated at 3.2385 Å. Then, following the equation of the a-lattice for a cubic crystal structure:

$$d(h,k,l) = \frac{a}{\sqrt{h^2+k^2+l^2}}, \tag{3}$$

with the Miller indices h, k, and l following the allowed reflection that gives a reflection

of XRD, and a the a-lattice of the cubic crystal structure. For the indices set at $h, k, l = 1$ in eq. 3, the a-lattice is determined as 5.61Å. By using the Brus equation[S1] detailed in the main manuscript, with the lattice constants $a_{Si} = 5.43071$ Å and $a_{\alpha-Sn} = 6.4912$ Å[S6], the Sn concentration is determined as about $16.9 \pm 0.3\,\%$.

Calculation of Sn concentration from FFT measurements. A similar method is used for the determination of Sn concentration. The FFT measurements provide directly the values of the d-spacing, with the possible diffractions allowed for specific sets of Miller indices, we can determine the a-lattice of a cubic crystal structure, and then the Sn concentration associated with the Brus equation[S1 S7].

Optimized structured. In the following we give the Cartesian coordinates of the optimized geometries of the nanocrystals as obtained from the density functional theory calculations.

Table S1: Cartesian coordinates of the optimized geometries of the nanocrystals Si_{268}, α-Sn_{268}, $Si_{222}Sn_{46}$(17% Sn-random), $Si_{222}Sn_{46}$(17% Sn-core), $Si_{222}Sn_{46}$(17% Sn-shell), and β-Sn_{268} as obtained from the density functional theory calculations.

Si_{268}				α-Sn_{268}			
Atom	x [Å]	y [Å]	z [Å]	Atom	x [Å]	y [Å]	z [Å]
Si	1.138258	9.230591	12.021208	Sn	1.147023	11.032920	14.340259
Si	1.071822	12.145530	14.938314	Sn	1.108502	14.376934	17.691282
Si	3.859454	3.859465	9.456111	Sn	4.442910	4.442908	11.111116
Si	2.501424	5.149511	10.937782	Sn	2.789468	6.073571	12.741965
Si	3.876146	6.626360	12.215836	Sn	4.410350	7.728713	14.397624
Si	2.495720	8.029802	13.568091	Sn	2.776711	9.423063	16.013543
Si	3.841811	9.418862	14.987290	Sn	4.383327	11.061814	17.715366

Si$_{268}$				α-Sn$_{268}$			
Si	2.460982	10.823677	16.356149	Sn	2.750338	12.752435	19.340058
Si	3.862432	12.172840	17.744487	Sn	4.417850	14.345330	21.007795
Si	5.149541	2.501417	10.937752	Sn	6.073574	2.789475	12.741977
Si	6.626377	3.876167	12.215823	Sn	7.728704	4.410367	14.397628
Si	5.280764	5.280760	13.596076	Sn	6.104514	6.104518	16.028790
Si	6.637496	6.637539	15.017913	Sn	7.730402	7.730393	17.728714
Si	5.262266	8.061894	16.370436	Sn	6.081050	9.428706	19.345637
Si	6.640652	9.428004	17.779601	Sn	7.712626	11.054890	21.045972
Si	5.263546	10.835210	19.145569	Sn	6.085265	12.747723	22.675210
Si	6.651858	12.184390	20.547041	Sn	7.753006	14.340618	24.342718
Si	9.230588	1.138290	12.021188	Sn	11.032909	1.147036	14.340276
Si	8.029811	2.495740	13.568082	Sn	9.423050	2.776722	16.013553
Si	9.418840	3.841861	14.987272	Sn	11.061828	4.383324	17.715364
Si	8.061865	5.262309	16.370416	Sn	9.428737	6.081059	19.345630
Si	9.427981	6.640663	17.779589	Sn	11.054900	7.712632	21.045975
Si	8.059223	8.059234	19.151053	Sn	9.415885	9.415887	22.664795
Si	9.421286	9.421318	20.587459	Sn	11.039451	11.039443	24.367678
Si	8.069623	10.862442	21.936170	Sn	9.401776	12.716111	25.984549
Si	12.145515	1.071860	14.938325	Sn	14.376956	1.108510	17.691290
Si	10.823639	2.461021	16.356137	Sn	12.752458	2.750337	19.340068
Si	12.172815	3.862485	17.744446	Sn	14.345343	4.417839	21.007811
Si	10.835191	5.263566	19.145560	Sn	12.747737	6.085274	22.675214
Si	12.184360	6.651896	20.547021	Sn	14.340641	7.753008	24.342722
Si	10.862419	8.069657	21.936156	Sn	12.716134	9.401785	25.984548
Si	1.138307	12.021188	9.230584	Sn	1.147038	14.340284	11.032888
Si	1.071823	14.938384	12.145560	Sn	1.108505	17.691280	14.376942

Si$_{268}$				α-Sn$_{268}$			
Si	3.841012	6.666852	6.666846	Sn	4.436604	7.787096	7.787077
Si	2.470185	8.054068	8.054019	Sn	2.782908	9.424431	9.424433
Si	3.878552	9.416953	9.416969	Sn	4.427543	11.068591	11.068590
Si	2.468125	10.788569	10.788587	Sn	2.766698	12.720334	12.720310
Si	3.825735	12.199057	12.199026	Sn	4.387605	14.396345	14.396329
Si	2.420518	13.586749	13.586729	Sn	2.725361	16.053621	16.053625
Si	3.856897	14.948837	14.948822	Sn	4.428260	17.677167	17.677165
Si	6.666826	3.841022	6.666826	Sn	7.787100	4.436601	7.787090
Si	5.224407	5.224409	8.017153	Sn	6.066131	6.066123	9.399126
Si	6.622030	6.622050	9.402190	Sn	7.724767	7.724750	11.057159
Si	5.248619	8.012334	10.804010	Sn	6.063772	9.384599	12.730696
Si	6.630273	9.408144	12.200498	Sn	7.718015	11.053265	14.396932
Si	5.229015	10.803915	13.585453	Sn	6.044467	12.718740	16.058343
Si	6.626816	12.194454	14.979666	Sn	7.708162	14.383204	17.722347
Si	5.228374	13.580074	16.367374	Sn	6.047083	16.038165	19.380438
Si	6.637547	14.946182	17.745742	Sn	7.747438	17.664342	21.012000
Si	8.054090	2.470212	8.053957	Sn	9.424457	2.782911	9.424434
Si	9.416958	3.878578	9.416939	Sn	11.068618	4.427537	11.068602
Si	8.012338	5.248641	10.803980	Sn	9.384613	6.063768	12.730703
Si	9.408115	6.630318	12.200480	Sn	11.053295	7.718005	14.396942
Si	8.021155	8.021184	13.605392	Sn	9.383860	9.383844	16.062879
Si	9.418278	9.418304	14.988315	Sn	11.048200	11.048193	17.726089
Si	8.028333	10.813620	16.381146	Sn	9.370741	12.709858	19.384919
Si	9.422515	12.204154	17.778990	Sn	11.034731	14.374346	21.048587
Si	8.020695	13.589223	19.166204	Sn	9.377693	16.031255	22.709738
Si	9.439919	14.978282	20.512265	Sn	11.079525	17.670000	24.316345

Si_{268}				α-Sn_{268}			
Si	12.021189	1.138270	9.230557	Sn	14.340282	1.147039	11.032905
Si	10.788566	2.468127	10.788545	Sn	12.720338	2.766692	12.720333
Si	12.199035	3.825760	12.199000	Sn	14.396357	4.387624	14.396331
Si	10.803895	5.229057	13.585437	Sn	12.718763	6.044466	16.058362
Si	12.194434	6.626873	14.979649	Sn	14.383232	7.708143	17.722359
Si	10.813594	8.028359	16.381150	Sn	12.709870	9.370726	19.384918
Si	12.204107	9.422558	17.778994	Sn	14.374348	11.034722	21.048583
Si	10.808956	10.808999	19.182257	Sn	12.696719	12.696708	22.705426
Si	12.219443	12.219469	20.539836	Sn	14.372730	14.372735	24.326345
Si	10.986796	13.777429	21.869681	Sn	12.752787	16.060130	25.946023
Si	14.938336	1.071855	12.145519	Sn	17.691294	1.108508	14.376945
Si	13.586706	2.420548	13.586703	Sn	16.053630	2.725388	16.053622
Si	14.948777	3.856964	14.948778	Sn	17.677182	4.428260	17.677180
Si	13.580049	5.228440	16.367360	Sn	16.038207	6.047070	19.380469
Si	14.946162	6.637603	17.745754	Sn	17.664368	7.747438	21.012025
Si	13.589199	8.020754	19.166209	Sn	16.031259	9.377707	22.709734
Si	14.978258	9.439980	20.512269	Sn	17.670021	11.079546	24.316343
Si	13.777385	10.986815	21.869716	Sn	16.060144	12.752811	25.946017
Si	3.859449	9.456092	3.859431	Sn	4.442907	11.111122	4.442909
Si	2.501447	10.937764	5.149483	Sn	2.789472	12.741980	6.073577
Si	3.876175	12.215810	6.626341	Sn	4.410375	14.397629	7.728706
Si	2.495727	13.568072	8.029749	Sn	2.776737	16.013566	9.423049
Si	3.841787	14.987305	9.418791	Sn	4.383332	17.715382	11.061812
Si	2.460983	16.356160	10.823640	Sn	2.750345	19.340064	12.752461
Si	3.862430	17.744491	12.172815	Sn	4.417857	21.007799	14.345338
Si	6.666810	6.666843	3.840983	Sn	7.787104	7.787088	4.436597

Si$_{268}$				α-Sn$_{268}$			
Si	5.224407	8.017184	5.224403	Sn	6.066139	9.399135	6.066117
Si	6.622037	9.402232	6.622046	Sn	7.724761	11.057152	7.724750
Si	5.248611	10.804005	8.012355	Sn	6.063773	12.730689	9.384597
Si	6.630261	12.200548	9.408104	Sn	7.718010	14.396927	11.053260
Si	5.228993	13.585495	10.803866	Sn	6.044470	16.058344	12.718732
Si	6.626782	14.979721	12.194406	Sn	7.708150	17.722339	14.383210
Si	5.228366	16.367406	13.580070	Sn	6.047095	19.380439	16.038193
Si	6.637559	17.745786	14.946166	Sn	7.747460	21.012013	17.664349
Si	9.456057	3.859441	3.859454	Sn	11.111140	4.442906	4.442904
Si	8.017141	5.224433	5.224393	Sn	9.399150	6.066126	6.066122
Si	9.402194	6.622076	6.622006	Sn	11.057178	7.724748	7.724735
Si	8.015210	8.015240	8.015206	Sn	9.387004	9.386989	9.386993
Si	9.409706	9.409757	9.409703	Sn	11.051085	11.051059	11.051051
Si	8.019960	10.805294	10.805239	Sn	9.376587	12.716851	12.716851
Si	9.414460	12.198278	12.198214	Sn	11.043570	14.382896	14.382897
Si	8.019620	13.589789	13.589726	Sn	9.366959	16.044870	16.044872
Si	9.402558	14.986895	14.986855	Sn	11.030218	17.709217	17.709224
Si	7.990058	16.370559	16.370534	Sn	9.364381	19.362667	19.362679
Si	9.411907	17.727333	17.727293	Sn	11.064296	20.988538	20.988551
Si	10.937723	2.501424	5.149542	Sn	12.741980	2.789481	6.073570
Si	12.215798	3.876208	6.626334	Sn	14.397647	4.410369	7.728699
Si	10.803980	5.248674	8.012318	Sn	12.730720	6.063771	9.384604
Si	12.200489	6.630323	9.408121	Sn	14.396955	7.718015	11.053275
Si	10.805254	8.020046	10.805256	Sn	12.716877	9.376580	12.716863
Si	12.198241	9.414560	12.198239	Sn	14.382918	11.043578	14.382904
Si	10.809764	10.809841	13.593494	Sn	12.710202	12.710193	16.049517

Si$_{268}$				α-Sn$_{268}$			
Si	12.202713	12.202809	14.988043	Sn	14.376239	14.376232	17.716512
Si	10.807448	13.599937	16.377750	Sn	12.696149	16.039805	19.375075
Si	12.203973	14.995720	17.759395	Sn	14.362358	17.708463	21.029309
Si	10.792178	16.381726	19.131860	Sn	12.695442	19.364363	22.682726
Si	12.070290	17.858508	20.506618	Sn	14.351122	21.019482	24.303594
Si	13.568049	2.495764	8.029762	Sn	16.013562	2.776717	9.423042
Si	14.987266	3.841835	9.418808	Sn	17.715382	4.383322	11.061814
Si	13.585469	5.229062	10.803874	Sn	16.058374	6.044470	12.718739
Si	14.979697	6.626870	12.194400	Sn	17.722350	7.708146	14.383218
Si	13.589761	8.019696	13.589719	Sn	16.044852	9.366958	16.044865
Si	14.986841	9.402633	14.986866	Sn	17.709228	11.030213	17.709216
Si	13.599844	10.807517	16.377728	Sn	16.039805	12.696135	19.375067
Si	14.995648	12.204017	17.759382	Sn	17.708450	14.362378	21.029289
Si	13.591048	13.591106	19.129431	Sn	16.024460	16.024483	22.665516
Si	14.953935	14.954015	20.537861	Sn	17.668647	17.668620	24.310156
Si	16.356127	2.461018	10.823638	Sn	19.340079	2.750332	12.752453
Si	17.744445	3.862472	12.172825	Sn	21.007806	4.417839	14.345348
Si	16.367373	5.228438	13.580059	Sn	19.380458	6.047080	16.038199
Si	17.745734	6.637627	14.946174	Sn	21.012031	7.747435	17.664364
Si	16.370508	7.990132	16.370535	Sn	19.362672	9.364365	19.362662
Si	17.727300	9.411956	17.727306	Sn	20.988546	11.064275	20.988551
Si	16.381684	10.792243	19.131852	Sn	19.364358	12.695457	22.682697
Si	17.858539	12.070314	20.506594	Sn	21.019494	14.351107	24.303596
Si	5.149486	10.937790	2.501424	Sn	6.073577	12.741972	2.789470
Si	6.626357	12.215844	3.876141	Sn	7.728711	14.397627	4.410349
Si	5.280727	13.596104	5.280714	Sn	6.104518	16.028776	6.104501

Si_{268}				α-Sn_{268}			
Si	6.637471	15.017964	6.637469	Sn	7.730387	17.728717	7.730392
Si	5.262251	16.370461	8.061855	Sn	6.081045	19.345643	9.428702
Si	6.640604	17.779655	9.427959	Sn	7.712618	21.046001	11.054888
Si	5.263523	19.145610	10.835201	Sn	6.085275	22.675216	12.747747
Si	6.651860	20.547051	12.184389	Sn	7.753017	24.342721	14.340635
Si	8.053998	8.054069	2.470168	Sn	9.424448	9.424429	2.782913
Si	9.416942	9.416982	3.878541	Sn	11.068601	11.068613	4.427521
Si	8.012340	10.804031	5.248626	Sn	9.384611	12.730703	6.063768
Si	9.408142	12.200547	6.630262	Sn	11.053278	14.396954	7.717994
Si	8.021128	13.605432	8.021108	Sn	9.383847	16.062886	9.383840
Si	9.418227	14.988374	9.418241	Sn	11.048209	17.726094	11.048202
Si	8.028298	16.381209	10.813575	Sn	9.370713	19.384918	12.709846
Si	9.422528	17.779007	12.204114	Sn	11.034710	21.048587	14.374343
Si	8.020735	19.166231	13.589217	Sn	9.377712	22.709746	16.031258
Si	9.439953	20.512327	14.978265	Sn	11.079545	24.316340	17.670021
Si	10.937745	5.149461	2.501428	Sn	12.741973	6.073570	2.789476
Si	12.215797	6.626362	3.876118	Sn	14.397635	7.728716	4.410354
Si	10.804006	8.012371	5.248595	Sn	12.730708	9.384622	6.063758
Si	12.200513	9.408157	6.630263	Sn	14.396951	11.053272	7.718013
Si	10.805268	10.805291	8.019977	Sn	12.716867	12.716870	9.376570
Si	12.198235	12.198300	9.414481	Sn	14.382917	14.382897	11.043566
Si	10.809740	13.593548	10.809753	Sn	12.710192	16.049503	12.710186
Si	12.202729	14.988038	12.202746	Sn	14.376228	17.716521	14.376215
Si	10.807475	16.377747	13.599881	Sn	12.696137	19.375069	16.039801
Si	12.203977	17.759405	14.995686	Sn	14.362359	21.029303	17.708461
Si	10.792195	19.131886	16.381693	Sn	12.695448	22.682707	19.364370

Si_{268}				α-Sn_{268}			
Si	12.070293	20.506582	17.858559	Sn	14.351127	24.303595	21.019488
Si	13.596071	5.280772	5.280712	Sn	16.028790	6.104505	6.104487
Si	15.017905	6.637526	6.637494	Sn	17.728730	7.730382	7.730377
Si	13.605395	8.021185	8.021149	Sn	16.062899	9.383832	9.383828
Si	14.988315	9.418317	9.418277	Sn	17.726105	11.048190	11.048210
Si	13.593512	10.809856	10.809788	Sn	16.049541	12.710184	12.710183
Si	14.988053	12.202819	12.202762	Sn	17.716540	14.376227	14.376216
Si	13.598286	13.598333	13.598316	Sn	16.041992	16.041987	16.041965
Si	14.992789	14.992834	14.992825	Sn	17.706043	17.706067	17.706051
Si	13.605775	16.386008	16.385994	Sn	16.035899	19.368290	19.368290
Si	14.990856	17.783603	17.783621	Sn	17.693907	21.026912	21.026927
Si	13.551978	19.148562	19.148621	Sn	15.981956	22.650137	22.650146
Si	16.370414	5.262282	8.061842	Sn	19.345644	6.081049	9.428714
Si	17.779599	6.640629	9.427959	Sn	21.046005	7.712613	11.054901
Si	16.381178	8.028358	10.813550	Sn	19.384928	9.370713	12.709863
Si	17.778972	9.422567	12.204113	Sn	21.048593	11.034713	14.374347
Si	16.377723	10.807516	13.599890	Sn	19.375071	12.696128	16.039795
Si	17.759399	12.204008	14.995693	Sn	21.029293	14.362363	17.708451
Si	16.385964	13.605801	16.385978	Sn	19.368272	16.035890	19.368260
Si	17.783579	14.990885	17.783597	Sn	21.026898	17.693858	21.026884
Si	16.341176	16.341189	19.167040	Sn	19.305967	19.305942	22.656440
Si	19.145557	5.263577	10.835215	Sn	22.675226	6.085252	12.747751
Si	20.547004	6.651914	12.184397	Sn	24.342731	7.753000	14.340632
Si	19.166179	8.020785	13.589219	Sn	22.709746	9.377694	16.031252
Si	20.512293	9.439964	14.978267	Sn	24.316338	11.079541	17.670017
Si	19.131887	10.792231	16.381713	Sn	22.682695	12.695443	19.364362

Si_{268}				α-Sn_{268}			
Si	20.506599	12.070305	17.858582	Sn	24.303587	14.351102	21.019494
Si	19.148576	13.552021	19.148591	Sn	22.650133	15.981947	22.650128
Si	9.230602	12.021229	1.138235	Sn	11.032920	14.340286	1.147032
Si	8.029770	13.568103	2.495691	Sn	9.423052	16.013547	2.776711
Si	9.418803	14.987323	3.841784	Sn	11.061813	17.715374	4.383317
Si	8.061836	16.370474	5.262246	Sn	9.428721	19.345642	6.081061
Si	9.427935	17.779673	6.640612	Sn	11.054909	21.045991	7.712638
Si	8.059175	19.151109	8.059199	Sn	9.415905	22.664822	9.415897
Si	9.421267	20.587482	9.421277	Sn	11.039461	24.367705	11.039456
Si	8.069658	21.936187	10.862470	Sn	9.401776	25.984557	12.716122
Si	12.021201	9.230634	1.138243	Sn	14.340274	11.032919	1.147025
Si	10.788560	10.788591	2.468111	Sn	12.720342	12.720341	2.766693
Si	12.199025	12.199055	3.825724	Sn	14.396359	14.396338	4.387626
Si	10.803891	13.585513	5.228992	Sn	12.718748	16.058372	6.044455
Si	12.194412	14.979748	6.626794	Sn	14.383207	17.722345	7.708156
Si	10.813542	16.381214	8.028298	Sn	12.709877	19.384922	9.370735
Si	12.204111	17.778996	9.422508	Sn	14.374349	21.048605	11.034721
Si	10.808962	19.182264	10.808948	Sn	12.696708	22.705435	12.696726
Si	12.219458	20.539818	12.219439	Sn	14.372737	24.326340	14.372747
Si	10.986823	21.869720	13.777373	Sn	12.752799	25.946016	16.060140
Si	13.568065	8.029799	2.495707	Sn	16.013548	9.423059	2.776711
Si	14.987274	9.418842	3.841802	Sn	17.715382	11.061806	4.383313
Si	13.585468	10.803920	5.229001	Sn	16.058373	12.718730	6.044460
Si	14.979680	12.194466	6.626809	Sn	17.722346	14.383206	7.708150
Si	13.589732	13.589795	8.019615	Sn	16.044862	16.044860	9.366958
Si	14.986846	14.986882	9.402594	Sn	17.709236	17.709234	11.030204

Si$_{268}$				α-Sn$_{268}$			
Si	13.599860	16.377742	10.807486	Sn	16.039818	19.375079	12.696123
Si	14.995664	17.759379	12.204006	Sn	17.708455	21.029324	14.362356
Si	13.591081	19.129444	13.591090	Sn	16.024470	22.665516	16.024487
Si	14.953982	20.537863	14.954008	Sn	17.668650	24.310142	17.668624
Si	16.370413	8.061906	5.262294	Sn	19.345645	9.428721	6.081051
Si	17.779588	9.428015	6.640668	Sn	21.045998	11.054909	7.712641
Si	16.381152	10.813646	8.028350	Sn	19.384931	12.709873	9.370732
Si	17.778997	12.204166	9.422564	Sn	21.048591	14.374358	11.034733
Si	16.377758	13.599959	10.807504	Sn	19.375079	16.039817	12.696138
Si	17.759418	14.995747	12.204020	Sn	21.029334	17.708456	14.362371
Si	16.385959	16.385992	13.605821	Sn	19.368285	19.368276	16.035872
Si	17.783563	17.783642	14.990879	Sn	21.026900	21.026900	17.693875
Si	16.341158	19.167056	16.341233	Sn	19.305962	22.656430	19.305956
Si	19.151039	8.059213	8.059213	Sn	22.664808	9.415890	9.415888
Si	20.587441	9.421289	9.421288	Sn	24.367677	11.039455	11.039466
Si	19.182262	10.808991	10.808979	Sn	22.705424	12.696719	12.696719
Si	20.539828	12.219472	12.219474	Sn	24.326364	14.372716	14.372722
Si	19.129454	13.591119	13.591105	Sn	22.665543	16.024455	16.024464
Si	20.537842	14.954017	14.954092	Sn	24.310150	17.668626	17.668643
Si	19.166996	16.341250	16.341212	Sn	22.656422	19.305953	19.305944
Si	21.936134	8.069667	10.862449	Sn	25.984557	9.401783	12.716115
Si	21.869697	10.986852	13.777434	Sn	25.946028	12.752798	16.060149
Si	12.145539	14.938362	1.071838	Sn	14.376959	17.691283	1.108501
Si	10.823657	16.356178	2.460989	Sn	12.752459	19.340059	2.750333
Si	12.172836	17.744489	3.862451	Sn	14.345340	21.007795	4.417845
Si	10.835211	19.145600	5.263529	Sn	12.747753	22.675205	6.085264

Si_{268}				α-Sn_{268}			
Si	12.184373	20.547062	6.651859	Sn	14.340642	24.342717	7.752998
Si	10.862445	21.936181	8.069659	Sn	12.716138	25.984555	9.401777
Si	14.938301	12.145548	1.071841	Sn	17.691293	14.376945	1.108503
Si	13.586730	13.586763	2.420540	Sn	16.053628	16.053617	2.725377
Si	14.948826	14.948839	3.856920	Sn	17.677176	17.677186	4.428258
Si	13.580093	16.367421	5.228388	Sn	16.038192	19.380451	6.047082
Si	14.946168	17.745788	6.637595	Sn	17.664360	21.012024	7.747428
Si	13.589201	19.166241	8.020729	Sn	16.031266	22.709742	9.377690
Si	14.978261	20.512296	9.439952	Sn	17.670014	24.316351	11.079526
Si	13.777403	21.869711	10.986812	Sn	16.060129	25.946008	12.752805
Si	16.356125	10.823682	2.460990	Sn	19.340073	12.752448	2.750335
Si	17.744469	12.172831	3.862437	Sn	21.007799	14.345343	4.417849
Si	16.367405	13.580089	5.228387	Sn	19.380460	16.038194	6.047082
Si	17.745749	14.946197	6.637597	Sn	21.012030	17.664353	7.747444
Si	16.370505	16.370541	7.990091	Sn	19.362679	19.362675	9.364361
Si	17.727271	17.727317	9.411933	Sn	20.988542	20.988537	11.064293
Si	16.381657	19.131864	10.792222	Sn	19.364373	22.682727	12.695438
Si	17.858477	20.506602	12.070301	Sn	21.019495	24.303597	14.351102
Si	19.145551	10.835234	5.263575	Sn	22.675217	12.747745	6.085263
Si	20.547000	12.184420	6.651904	Sn	24.342727	14.340628	7.753006
Si	19.166195	13.589263	8.020775	Sn	22.709748	16.031254	9.377704
Si	20.512278	14.978320	9.439997	Sn	24.316356	17.670015	11.079543
Si	19.131858	16.381771	10.792221	Sn	22.682725	19.364372	12.695446
Si	20.506571	17.858602	12.070348	Sn	24.303598	21.019499	14.351119
Si	19.148515	19.148653	13.552016	Sn	22.650134	22.650148	15.981947
Si	21.936128	10.862453	8.069645	Sn	25.984550	12.716120	9.401781

Si$_{268}$				α-Sn$_{268}$			
Si	21.869704	13.777405	10.986824	Sn	25.946031	16.060139	12.752803
H	0.586952	8.199200	10.996091	H	0.169601	9.980477	13.293608
H	0.000000	9.962083	12.737881	H	0.082750	12.032821	15.348271
H	0.269707	11.215444	14.010332	H	0.144074	13.322423	16.638723
H	0.026049	12.969127	15.742011	H	0.000000	15.352819	18.679416
H	2.945431	2.945453	8.552668	H	3.396285	3.396281	10.096496
H	1.418850	5.968035	10.232803	H	1.796785	7.123796	11.718118
H	1.796778	4.185668	11.927614	H	1.700839	5.074059	13.728106
H	1.599542	7.089415	14.462213	H	1.723752	8.395477	17.041464
H	1.585166	9.872917	17.258392	H	1.708606	11.722748	20.376945
H	2.978212	13.124412	18.637812	H	3.384930	15.381720	22.048346
H	4.185663	1.796772	11.927617	H	5.074064	1.700846	13.728110
H	5.967988	1.418888	10.232751	H	7.123795	1.796793	11.718126
H	4.349515	4.349484	14.461408	H	5.063616	5.063618	17.058475
H	4.345956	7.165598	17.291617	H	5.042817	8.388697	20.380526
H	4.370247	9.883638	20.029805	H	5.044709	11.711332	23.708127
H	5.749591	13.135138	21.422844	H	6.716106	15.370287	25.384459
H	9.962063	0.000000	12.737866	H	12.032819	0.082768	15.348283
H	8.199165	0.587042	10.996085	H	9.980490	0.169609	13.293612
H	7.089409	1.599581	14.462199	H	8.395472	1.723753	17.041469
H	7.165600	4.346015	17.291637	H	8.388707	5.042842	20.380511
H	7.140744	7.140792	20.048083	H	8.376931	8.376928	23.698481
H	8.997734	11.792600	22.738291	H	10.454326	13.770629	26.948994
H	7.266050	10.038937	22.981958	H	8.413643	11.740224	27.093045
H	12.969079	0.026082	15.741990	H	15.352838	0.000002	18.679419
H	11.215409	0.269752	14.010292	H	13.322438	0.144080	16.638722

Si_{268}				α-Sn_{268}			
H	9.872899	1.585211	17.258407	H	11.722762	1.708610	20.376948
H	13.124389	2.978283	18.637778	H	15.381734	3.384925	22.048368
H	9.883611	4.370249	20.029772	H	11.711348	5.044720	23.708134
H	13.135110	5.749637	21.422832	H	15.370305	6.716103	25.384462
H	11.792527	8.997712	22.738339	H	13.770642	10.454344	26.948989
H	10.038843	7.266034	22.981884	H	11.740243	8.413647	27.093042
H	0.586877	10.996174	8.199242	H	0.169615	13.293620	9.980465
H	0.000039	12.737925	9.962117	H	0.082769	15.348280	12.032811
H	0.269706	14.010332	11.215469	H	0.144076	16.638727	13.322421
H	0.026020	15.742036	12.969085	H	0.000006	18.679419	15.352825
H	2.923671	5.759108	5.759062	H	3.389805	6.755479	6.755476
H	1.575029	8.989189	7.226832	H	1.780382	10.461337	8.390500
H	1.575030	7.226835	8.989241	H	1.780383	8.390497	10.461342
H	2.959851	15.867332	15.867273	H	3.394570	18.716123	18.716124
H	5.759049	2.923702	5.759044	H	6.755500	3.389801	6.755476
H	5.716348	15.842465	18.662047	H	6.712534	18.704345	22.050225
H	7.226807	1.575013	8.989193	H	8.390510	1.780399	10.461351
H	8.989116	1.575093	7.226739	H	10.461352	1.780381	8.390499
H	8.545846	15.918672	21.408486	H	10.051617	18.697583	25.369319
H	12.737867	0.000007	9.962029	H	15.348283	0.082766	12.032807
H	10.996094	0.586970	8.199135	H	13.293616	0.169618	9.980482
H	12.011884	14.808775	22.421074	H	13.799458	17.112544	26.923445
H	10.270162	13.045936	23.007950	H	11.744770	15.060228	27.010290
H	15.742020	0.026073	12.969037	H	18.679424	0.000000	15.352827
H	14.010284	0.269731	11.215400	H	16.638725	0.144077	13.322427
H	15.867239	2.959938	15.867249	H	18.716143	3.394577	18.716133

	Si$_{268}$				α-Sn$_{268}$		
H	15.842461	5.716426	18.662050	H	18.704379	6.712536	22.050249
H	15.918630	8.545875	21.408480	H	18.697592	10.051629	25.369311
H	14.808756	12.011909	22.421143	H	17.112565	13.799490	26.923435
H	13.045894	10.270179	23.007949	H	15.060247	11.744806	27.010296
H	2.945422	8.552649	2.945403	H	3.396281	10.096496	3.396278
H	1.796749	11.927574	4.185719	H	1.700839	13.728110	5.074057
H	1.418882	10.232692	5.968009	H	1.796794	11.718124	7.123801
H	1.599540	14.462170	7.089359	H	1.723767	17.041481	8.395459
H	1.585165	17.258418	9.872894	H	1.708619	20.376946	11.722758
H	2.978195	18.637801	13.124387	H	3.384934	22.048347	15.381730
H	5.759039	5.759069	2.923657	H	6.755495	6.755480	3.389801
H	5.716369	18.662121	15.842420	H	6.712542	22.050238	18.704351
H	8.552575	2.945446	2.945441	H	10.096498	3.396290	3.396286
H	8.546684	18.658652	18.658600	H	10.034626	22.029441	22.029449
H	10.232768	1.418886	5.968053	H	11.718137	1.796806	7.123801
H	11.927542	1.796737	4.185693	H	13.728126	1.700840	5.074060
H	11.080460	18.822284	21.211338	H	13.364970	22.018996	25.392219
H	12.775285	17.039985	21.589180	H	15.374969	19.969269	25.296268
H	14.462186	1.599608	7.089379	H	17.041477	1.723748	8.395461
H	14.018820	15.781205	21.433028	H	16.631747	18.702563	25.312684
H	15.781128	14.018774	21.433015	H	18.702572	16.631698	25.312663
H	17.258422	1.585219	9.872905	H	20.376972	1.708608	11.722758
H	18.637799	2.978259	13.124368	H	22.048365	3.384927	15.381738
H	18.662053	5.716482	15.842492	H	22.050249	6.712528	18.704377
H	18.658561	8.546694	18.658628	H	22.029447	10.034606	22.029456
H	17.040030	12.775307	21.589178	H	19.969271	15.374942	25.296281

Si_{268}				α-Sn_{268}			
H	18.822327	11.080439	21.211239	H	22.019022	13.364956	25.392217
H	5.967988	10.232758	1.418840	H	7.123808	11.718117	1.796801
H	4.185667	11.927603	1.796757	H	5.074067	13.728108	1.700841
H	4.349409	14.461363	4.349445	H	5.063616	17.058467	5.063606
H	4.345949	17.291649	7.165572	H	5.042806	20.380535	8.388693
H	4.370205	20.029846	9.883643	H	5.044720	23.708136	11.711350
H	5.749606	21.422880	13.135137	H	6.716116	25.384457	15.370301
H	8.989183	7.226828	1.575007	H	10.461353	8.390497	1.780394
H	7.226798	8.989182	1.574999	H	8.390509	10.461342	1.780390
H	8.545881	21.408519	15.918697	H	10.051614	25.369320	18.697576
H	11.927540	4.185682	1.796713	H	13.728128	5.074063	1.700844
H	10.232700	5.967999	1.418848	H	11.718136	7.123801	1.796799
H	12.775257	21.589167	17.040069	H	15.374972	25.296265	19.969263
H	11.080403	21.211240	18.822340	H	13.364972	25.392225	22.018998
H	14.461356	4.349478	4.349449	H	17.058496	5.063614	5.063601
H	14.455531	20.062525	20.062602	H	16.996611	23.696747	23.696752
H	17.291624	4.346021	7.165543	H	20.380541	5.042825	8.388695
H	17.248930	17.248965	20.084371	H	20.337586	20.337554	23.703243
H	20.029781	4.370274	9.883651	H	23.708142	5.044711	11.711343
H	21.422829	5.749654	13.135130	H	25.384481	6.716108	15.370296
H	21.408506	8.545856	15.918637	H	25.369310	10.051619	18.697584
H	21.211239	11.080439	18.822370	H	25.392223	13.364955	22.019021
H	21.589189	12.775310	17.040077	H	25.296277	15.374935	19.969272
H	20.062538	14.455567	20.062577	H	23.696736	16.996610	23.696735
H	8.199169	10.996075	0.586947	H	9.980497	13.293621	0.169619
H	9.962048	12.737855	0.000000	H	12.032830	15.348292	0.082762

	Si$_{268}$				α-Sn$_{268}$		
H	7.089372	14.462209	1.599515	H	8.395466	17.041475	1.723752
H	7.165530	17.291660	4.345955	H	8.388719	20.380539	5.042822
H	7.140731	20.048192	7.140750	H	8.376945	23.698505	8.376925
H	7.265986	22.981998	10.038919	H	8.413637	27.093055	11.740234
H	8.997665	22.738317	11.792550	H	10.454330	26.948999	13.770632
H	10.996048	8.199261	0.586925	H	13.293609	9.980492	0.169614
H	12.737865	9.962104	0.000010	H	15.348278	12.032819	0.082750
H	10.270227	23.007977	13.045891	H	11.744778	27.010278	15.060233
H	12.011925	22.421133	14.808748	H	13.799465	26.923452	17.112546
H	14.462171	7.089401	1.599535	H	17.041474	8.395473	1.723756
H	15.781294	21.433004	14.018944	H	18.702576	25.312652	16.631710
H	14.018774	21.433114	15.781136	H	16.631745	25.312672	18.702552
H	17.291594	7.165644	4.345971	H	20.380543	8.388703	5.042826
H	17.248944	20.084374	17.249006	H	20.337581	23.703241	20.337560
H	20.048101	7.140782	7.140747	H	23.698498	8.376943	8.376929
H	21.432979	14.018965	15.781368	H	25.312664	16.631721	18.702580
H	21.433035	15.781352	14.018972	H	25.312672	18.702565	16.631739
H	20.084308	17.249043	17.248987	H	23.703239	20.337564	20.337567
H	22.738268	8.997746	11.792555	H	26.948994	10.454337	13.770646
H	22.981904	7.266038	10.038894	H	27.093033	8.413649	11.740221
H	22.421150	12.011896	14.808750	H	26.923440	13.799478	17.112565
H	23.007978	10.270192	13.045908	H	27.010309	11.744783	15.060256
H	11.215455	14.010340	0.269704	H	13.322429	16.638723	0.144072
H	12.969103	15.742039	0.026064	H	15.352841	18.679423	0.000002
H	9.872962	17.258478	1.585161	H	11.722768	20.376951	1.708602
H	13.124379	18.637844	2.978227	H	15.381741	22.048347	3.384925

Si$_{268}$				α-Sn$_{268}$			
H	9.883656	20.029834	4.370202	H	11.711353	23.708126	5.044718
H	13.135096	21.422913	5.749605	H	15.370310	25.384467	6.716100
H	10.038870	22.981997	7.266021	H	11.740239	27.093041	8.413642
H	11.792505	22.738309	8.997685	H	13.770641	26.948995	10.454336
H	14.010326	11.215499	0.269694	H	16.638723	13.322429	0.144073
H	15.742028	12.969149	0.026063	H	18.679429	15.352825	0.000000
H	15.867268	15.867320	2.959856	H	18.716142	18.716138	3.394571
H	15.842494	18.662096	5.716451	H	18.704372	22.050251	6.712523
H	15.918708	21.408482	8.545893	H	18.697588	25.369322	10.051610
H	13.045872	23.007924	10.270160	H	15.060227	27.010273	11.744785
H	14.808749	22.421163	12.011900	H	17.112551	26.923428	13.799466
H	17.258393	9.872944	1.585172	H	20.376966	11.722757	1.708604
H	18.637801	13.124375	2.978181	H	22.048355	15.381731	3.384927
H	18.662039	15.842530	5.716447	H	22.050245	18.704363	6.712531
H	18.658626	18.658619	8.546761	H	22.029448	22.029443	10.034635
H	18.822189	21.211356	11.080507	H	22.019022	25.392218	13.364954
H	17.039881	21.589196	12.775343	H	19.969275	25.296280	15.374942
H	20.029797	9.883675	4.370274	H	23.708138	11.711347	5.044712
H	21.422829	13.135146	5.749630	H	25.384468	15.370305	6.716105
H	21.408482	15.918699	8.545905	H	25.369317	18.697588	10.051617
H	21.589033	17.040020	12.775382	H	25.296264	19.969272	15.374968
H	21.211338	18.822385	11.080541	H	25.392231	22.019011	13.364969
H	20.062438	20.062645	14.455609	H	23.696744	23.696755	16.996607
H	22.738267	11.792554	8.997713	H	26.948994	13.770645	10.454328
H	22.981923	10.038926	7.266004	H	27.093030	11.740221	8.413640
H	22.421132	14.808763	12.011951	H	26.923443	17.112564	13.799475

Si$_{268}$				α-Sn$_{268}$			
H	23.007940	13.045931	10.270214	H	27.010309	15.060251	11.744781

Si$_{222}$Sn$_{46}$(17% Sn-random)				Si$_{222}$Sn$_{46}$(17% Sn-core)			
Atom	x [Å]	y [Å]	z [Å]	Atom	x [Å]	y [Å]	z [Å]
Sn	1.332452	9.500360	12.819815	Sn	6.748203	18.697562	9.908580
Si	1.885981	12.535481	15.598636	Sn	14.326191	5.259584	11.301690
Si	4.570100	3.579330	9.977456	Sn	12.802078	6.926818	9.707416
Sn	2.758543	4.888690	11.342385	Sn	12.656492	6.856433	4.050714
Sn	4.250759	6.821912	12.744852	Sn	12.660842	15.764570	18.751847
Si	2.926578	8.272129	14.467457	Sn	12.560133	3.880906	12.970928
Si	4.385939	9.545843	15.894644	Sn	5.451099	8.247582	11.418554
Si	3.129106	11.175484	17.141379	Sn	6.710338	12.610097	15.765009
Si	4.589109	12.513587	18.488013	Sn	5.121356	14.283389	17.389624
Si	6.057792	2.481949	11.483022	Sn	10.997763	2.263141	11.286418
Si	7.487730	3.994139	12.650426	Sn	3.721389	15.641047	9.804407
Sn	5.984673	5.306589	14.308295	Sn	11.097362	5.316850	8.250600
Si	7.491034	6.919252	15.689860	Sn	8.257915	14.253165	14.114696
Si	6.105547	8.314218	17.108448	Sn	14.085607	8.303743	14.275022
Si	7.469303	9.762534	18.486624	Sn	11.031841	14.273210	17.116882
Si	6.007941	11.114471	19.835407	Sn	12.766147	12.657399	15.708292
Si	7.290522	12.345847	21.440946	Sn	9.737352	15.647280	10.015517
Si	10.139088	1.338536	12.482308	Sn	8.167866	14.069338	8.367953
Si	8.907246	2.667520	14.018076	Sn	3.762019	9.798614	9.813171
Si	10.222045	4.036912	15.476458	Sn	8.299079	11.185675	5.521125

$Si_{222}Sn_{46}$(17% Sn-random)				$Si_{222}Sn_{46}$(17% Sn-core)			
Si	8.805189	5.346940	16.921813	Sn	6.609572	15.774742	6.726115
Sn	10.241403	6.719977	18.596169	Sn	3.795099	9.718470	15.732789
Si	8.805088	8.419301	19.966542	Sn	5.242578	17.166299	14.259594
Si	10.250481	9.813710	21.289614	Sn	11.131455	8.279584	11.418784
Si	8.888109	11.154310	22.726426	Sn	11.208360	11.235086	2.531098
Si	13.136951	1.017539	15.250845	Sn	6.658239	12.817554	3.887559
Sn	11.511548	2.229102	16.875942	Sn	17.012238	8.275560	11.241596
Sn	13.143998	3.709691	18.598477	Sn	8.073388	14.313470	20.297260
Si	11.689400	5.202274	20.157370	Sn	17.114275	8.103762	17.286672
Sn	13.047036	6.842398	21.635096	Sn	16.948810	14.127039	11.225080
Si	11.546705	8.570005	22.864173	Sn	9.546823	12.534820	18.673520
Si	1.598194	12.637359	9.617523	Sn	17.050914	11.182725	20.135133
Si	1.738080	15.319419	12.719998	Sn	6.730133	6.758700	15.813687
Si	4.386628	6.699383	6.969674	Sn	19.983363	11.219360	11.315225
Sn	2.938144	8.283124	8.438372	Sn	13.969588	17.025995	11.313185
Si	4.624683	9.703857	9.847076	Sn	17.032921	17.072098	14.329589
Sn	2.990784	11.189932	11.281567	Sn	15.638090	6.844590	6.932779
Si	4.472215	12.723526	12.797007	Sn	11.049069	11.134907	14.170941
Si	3.121469	14.083945	14.238717	Sn	17.067827	17.091837	8.257240
Si	4.651773	15.363695	15.579388	Sn	15.534676	12.695219	6.937571
Si	7.737249	3.861171	6.965346	Sn	16.873091	11.224400	14.266448
Sn	5.951297	5.152297	8.389447	Sn	9.540326	18.854121	6.593294
Si	7.388708	6.816829	9.863259	Sn	9.614575	9.910712	9.760260
Si	5.898070	8.174513	11.188596	Sn	18.537580	9.705219	6.878127
Sn	7.471659	9.608522	12.699763	Sn	14.156565	20.142730	8.350050
Sn	5.810391	11.109542	14.350569	Sn	11.164356	17.223331	8.224238

$Si_{222}Sn_{46}$(17% Sn-random)				$Si_{222}Sn_{46}$(17% Sn-core)			
Si	7.390102	12.532696	15.850247	Si	4.062429	6.913752	7.053111
Si	5.923150	13.961508	17.110149	Si	19.982933	8.184246	8.466772
Si	7.212245	15.369254	18.532273	Si	6.897999	4.184092	7.022858
Si	9.044141	2.661172	8.569548	Si	3.692404	15.572213	15.634923
Si	10.282782	4.178338	9.942446	Si	2.429382	8.170068	8.282048
Si	8.818696	5.509822	11.327106	Si	2.429597	8.212701	14.075047
Si	10.280806	6.826604	12.766123	Si	1.184085	12.668878	15.545708
Si	8.977923	8.192428	14.280124	Si	4.181130	4.091914	9.921759
Si	10.299775	9.697054	15.656676	Si	2.694865	5.319494	11.347841
Si	8.903377	11.110273	17.089232	Si	2.400225	11.402243	17.180766
Si	10.334005	12.519536	18.448145	Si	14.079403	5.285413	5.554544
Sn	8.788359	13.959723	20.037095	Si	14.179790	22.546587	11.616204
Si	10.444813	15.468238	21.388806	Si	12.621551	15.442196	1.438385
Si	12.864766	1.371516	9.772661	Si	15.642520	21.387724	10.114310
Si	11.660640	2.752506	11.305847	Si	15.441183	18.475169	6.782349
Si	13.095740	4.045618	12.754655	Si	15.513972	15.569463	4.139130
Si	11.703072	5.438604	14.165846	Si	15.419289	12.641979	1.431531
Si	13.091281	6.816939	15.627842	Si	11.214267	22.730122	8.563505
Si	11.726599	8.269579	17.046996	Si	12.494211	21.553129	6.899009
Si	13.106251	9.664300	18.475829	Si	11.436912	20.085015	5.289118
Si	11.709778	11.117649	19.856344	Si	12.701966	18.345012	4.151031
Si	13.058017	12.565862	21.261542	Si	11.312198	16.925815	2.784968
Si	11.886133	14.092598	22.677918	Si	5.470136	2.716366	11.408935
Si	15.877004	1.212817	12.668100	Si	22.541296	11.423618	14.419501
Si	14.507011	2.582468	14.066238	Si	22.652078	8.361265	11.423461
Si	15.838974	4.029087	15.455217	Si	3.849991	12.482591	18.788058

$Si_{222}Sn_{46}$(17% Sn-random)				$Si_{222}Sn_{46}$(17% Sn-core)			
Si	14.435565	5.290501	16.941935	Si	11.232446	2.654076	17.033325
Sn	15.976862	6.760094	18.441212	Si	5.334023	5.309006	14.173860
Si	14.447486	8.270096	19.935976	Si	1.113766	12.470149	9.812042
Sn	15.934201	9.668960	21.568411	Si	18.593948	9.832776	18.538247
Si	14.520875	11.471267	22.793339	Si	18.538793	6.779963	15.547659
Sn	4.334621	9.788145	3.803842	Si	18.331966	4.037629	12.734073
Si	2.923305	11.356054	5.334518	Si	2.671556	11.227531	5.532149
Sn	4.469625	12.624761	7.004729	Si	21.067178	15.583600	15.678983
Si	3.073702	14.158659	8.552870	Si	2.424005	13.863342	8.370045
Si	4.584017	15.393895	9.940092	Si	19.726126	14.128208	19.784694
Si	3.056489	16.694277	11.270140	Si	8.297512	20.227657	8.464461
Sn	4.424880	18.368641	12.700404	Si	9.501608	12.688318	1.195802
Si	7.655586	6.757927	4.178967	Si	8.234115	2.754710	14.241578
Sn	5.944472	8.210098	5.517694	Si	1.175598	15.453406	12.763441
Si	7.444611	9.720150	7.099600	Si	11.207871	8.363805	22.724006
Si	6.042139	11.114616	8.498426	Si	5.319631	8.316836	17.338615
Si	7.404934	12.562100	9.921214	Si	12.522500	6.920398	21.338348
Si	5.955926	13.963571	11.335159	Si	11.229135	5.514391	19.885182
Si	7.323103	15.460679	12.649288	Si	12.547634	4.110275	18.426363
Si	5.953804	16.790543	14.127668	Si	12.594849	1.229817	15.665558
Si	7.162125	18.269275	15.563408	Si	8.431736	11.227577	22.704106
Si	10.367875	4.122978	4.145456	Si	8.401519	8.352609	19.960335
Si	9.014480	5.422701	5.644725	Si	8.449028	5.405653	17.233270
Si	10.287482	6.892142	7.095766	Si	9.286279	1.088986	12.869632
Si	8.810449	8.249941	8.485998	Si	16.929183	8.401272	5.290352
Si	10.261126	9.655978	9.855072	Si	6.713097	12.463782	21.561522

$Si_{222}Sn_{46}$(17% Sn-random)				$Si_{222}Sn_{46}$(17% Sn-core)			
Si	8.939377	11.170349	11.205797	Si	5.456205	11.121049	19.994784
Si	10.353126	12.452338	12.694217	Si	3.965595	12.615067	6.993084
Sn	8.814563	13.941599	14.184837	Si	12.672101	12.743133	4.116672
Si	10.379153	15.383362	15.694558	Si	12.524971	21.086891	18.427312
Sn	8.728475	16.890941	17.093400	Si	5.299673	11.172039	14.126474
Sn	10.284948	18.480865	18.761733	Si	11.162016	8.320487	17.168568
Sn	11.871672	2.502752	5.513487	Si	21.420707	9.611410	9.790842
Si	13.096712	4.102011	7.184106	Si	11.121658	19.883794	11.327826
Si	11.686860	5.532210	8.528226	Si	18.483409	15.563586	12.777935
Si	13.101277	6.903131	9.948897	Si	8.402567	19.868294	14.221058
Si	11.646304	8.276479	11.353338	Si	12.647187	12.724598	9.838710
Si	13.020684	9.563818	12.890339	Si	19.778145	14.157416	14.246899
Si	11.694608	11.093852	14.216679	Si	12.725942	3.806813	6.847976
Si	13.132151	12.513737	15.610806	Si	5.476309	5.508211	8.449145
Si	11.750878	13.932131	17.058807	Si	11.291808	11.357486	8.340404
Si	13.153692	15.353313	18.461726	Si	12.604851	9.854352	6.926864
Si	11.846845	16.759031	19.940232	Si	8.289321	5.466262	11.356237
Si	13.138079	18.255861	21.277248	Si	15.444068	15.612070	9.696395
Si	14.440155	2.703463	8.590654	Si	13.958186	8.284672	19.968922
Si	15.857234	3.962682	10.050853	Si	6.953743	6.813170	9.841604
Si	14.475768	5.459465	11.349516	Si	9.740064	18.457663	12.777621
Si	15.896174	6.864213	12.715182	Si	9.891507	12.711543	6.931203
Si	14.512738	8.197946	14.206456	Si	9.863028	15.533480	4.111340
Si	15.906505	9.684414	15.556907	Si	9.813281	9.642669	4.079120
Si	14.486440	11.018371	16.997493	Si	8.306018	8.306060	2.804292
Sn	16.058291	12.478844	18.510344	Si	9.581410	3.992412	9.937234

$Si_{222}Sn_{46}$(17% Sn-random)				$Si_{222}Sn_{46}$(17% Sn-core)			
Si	14.467702	13.982960	19.931291	Si	11.041464	17.281975	20.145513
Si	15.883955	15.331604	21.297396	Si	8.182698	17.132958	11.462432
Si	17.308899	2.621858	11.403383	Si	13.971718	11.202129	5.447058
Si	18.678970	4.081628	12.725029	Si	15.499719	18.525445	12.803413
Si	17.268730	5.448642	14.110393	Si	13.990366	14.191541	8.434268
Si	18.683297	6.846065	15.475475	Si	4.015733	9.751455	4.212764
Si	17.381759	8.290414	16.871494	Si	14.082439	16.931810	5.538006
Sn	18.978481	9.627033	18.450007	Si	8.299229	17.034429	5.174029
Si	17.587193	11.051451	20.121070	Si	15.319595	9.680514	21.404295
Sn	19.163007	12.592752	21.526118	Si	12.619451	1.101818	9.588505
Si	5.917188	11.312728	2.413095	Si	8.193851	2.759498	8.432063
Sn	7.376886	12.674773	4.083424	Si	6.825726	15.857401	18.584810
Si	6.038197	14.199645	5.688062	Si	1.227682	9.676420	12.595233
Si	7.472238	15.411922	7.161921	Si	11.159659	8.373360	5.606969
Si	6.018151	16.771170	8.540269	Si	18.533744	18.586504	15.851602
Si	7.370271	18.285338	9.846909	Si	21.307505	12.832090	12.924525
Si	6.040881	19.772630	11.199748	Si	14.121702	14.137750	2.778047
Si	7.333646	21.350847	12.465828	Si	17.035942	11.183373	8.454374
Si	8.952209	8.300995	2.907882	Si	17.063035	14.220223	5.428261
Si	10.257515	9.695935	4.347270	Si	9.790018	21.380835	9.959051
Si	8.811256	11.117054	5.664814	Si	16.952608	19.848321	11.418360
Si	10.197536	12.528934	7.059832	Si	19.850693	14.062554	8.395307
Si	8.842405	13.942846	8.522284	Si	18.455467	12.627475	9.724362
Si	10.209031	15.398275	9.935624	Si	19.782952	16.963387	11.323231
Si	8.705318	16.813862	11.210021	Si	12.594010	21.172657	12.781394
Sn	10.206189	18.259867	12.785549	Si	14.023350	19.815075	14.182161

$Si_{222}Sn_{46}$(17% Sn-random)				$Si_{222}Sn_{46}$(17% Sn-core)			
Sn	8.625674	20.032543	14.322170	Si	18.367331	18.447522	10.064194
Si	10.420306	21.359280	15.726195	Si	11.251011	14.132923	5.527576
Si	11.847259	5.466320	2.846792	Si	12.673367	15.535352	6.911702
Si	13.097893	6.877323	4.315915	Si	12.579200	18.673195	9.848872
Si	11.696278	8.282908	5.693951	Si	15.451035	9.866536	4.077796
Si	13.122598	9.668491	7.078846	Si	18.614345	12.804496	21.444076
Si	11.591879	11.033530	8.365431	Si	19.703134	16.910420	17.138966
Sn	13.026842	12.630436	9.826659	Si	18.472898	21.106510	12.775022
Si	11.608456	14.090717	11.434658	Si	15.279549	6.782570	18.612390
Si	13.149570	15.392701	12.800682	Si	16.868109	14.084885	17.096077
Si	11.730245	16.717832	14.242996	Si	6.745431	9.724816	7.057064
Sn	13.166329	18.330472	15.689200	Si	3.890293	6.712849	12.877962
Si	11.727668	19.959218	17.177248	Si	15.538529	12.638351	18.541448
Si	13.147353	21.108600	18.719122	Si	2.556238	14.066894	14.158460
Si	14.419532	5.487005	5.754214	Si	14.152188	13.990613	19.978667
Si	15.832177	6.852550	7.083378	Si	8.217071	8.312842	14.282478
Si	14.521227	8.281663	8.520949	Si	9.645867	9.587514	15.756674
Si	16.062484	9.617399	9.839344	Si	8.181596	10.937513	17.135480
Sn	14.603342	11.037842	11.451150	Si	17.049269	5.357261	14.295446
Si	16.080468	12.630772	12.875662	Si	9.582188	6.824242	12.919727
Si	14.556861	13.961193	14.223898	Si	19.724781	11.295152	17.010562
Si	15.962797	15.385990	15.622392	Si	14.148801	11.109221	17.283871
Si	14.643785	16.741186	17.146860	Si	15.540384	9.707471	15.924542
Si	16.005325	18.170131	18.529035	Si	15.697997	6.758600	12.905144
Si	14.568008	19.558452	19.877492	Si	15.766179	3.772915	9.708095
Sn	17.407353	5.374065	8.520590	Si	11.003622	5.482549	14.366516

$Si_{222}Sn_{46}$(17% Sn-random)				$Si_{222}Sn_{46}$(17% Sn-core)			
Si	18.874212	6.916713	10.027804	Si	12.451020	6.862739	15.730271
Si	17.367720	8.255965	11.353983	Si	12.514193	15.463281	12.866145
Si	18.702713	9.641124	12.838013	Si	3.999184	12.708959	12.754269
Si	17.395365	11.131981	14.255696	Si	15.471323	15.483811	15.691776
Si	18.849840	12.565680	15.590910	Si	14.117118	17.018595	16.985959
Si	17.478345	14.024472	16.946322	Si	15.512356	18.399676	18.386501
Si	18.767261	15.420472	18.444576	Si	6.640445	12.616031	9.924938
Si	17.312109	16.680968	19.908381	Si	5.384211	11.237589	8.383582
Si	20.160734	5.441729	11.404324	Si	14.045283	11.288136	11.229821
Si	21.562227	6.860922	12.717877	Si	8.101757	11.326223	11.353043
Si	20.144830	8.198072	14.125346	Si	9.620795	6.983373	6.868733
Sn	21.771182	9.714108	15.538217	Si	11.255319	19.681655	16.972827
Si	20.334966	11.314883	17.015448	Si	19.908971	11.457996	5.504451
Si	21.632337	12.557926	18.588218	Si	8.268714	17.141837	17.111712
Si	20.303152	14.144221	19.778330	Si	9.647673	15.847277	15.615496
Si	10.076075	12.324974	1.473064	Si	15.363013	12.689744	12.701100
Si	9.098935	14.078954	2.759619	Si	11.165182	17.034353	14.173156
Si	10.307606	15.445100	4.317851	Si	14.133605	14.191019	14.139880
Si	8.896979	16.767428	5.773470	Si	15.500507	9.914663	9.813854
Si	10.304095	18.148488	7.140715	Si	2.465614	11.369961	11.441282
Si	8.924043	19.558231	8.506587	Si	12.609078	9.730805	18.568849
Si	10.296359	21.020693	9.856518	Si	12.681090	18.344770	15.551078
Si	9.002524	22.502547	11.219172	Si	9.700120	3.994695	15.735840
Si	12.859157	9.453085	1.522940	Si	12.684853	12.606637	21.307915
Si	11.556687	10.996251	2.799428	Si	5.319050	14.145745	11.276754
Sn	13.120531	12.582361	4.170084	Si	11.501050	14.218593	22.631571

$Si_{222}Sn_{46}$(17% Sn-random)				$Si_{222}Sn_{46}$(17% Sn-core)			
Si	11.618695	14.021083	5.754506	Si	15.434792	1.308489	12.771577
Si	13.093460	15.413437	7.081724	Si	12.731796	9.849025	12.669535
Si	11.704032	16.773013	8.558559	Si	9.780813	6.914745	18.572216
Si	13.117131	18.215342	9.914822	Si	11.262969	11.117426	20.029251
Si	11.755047	19.697172	11.248337	Si	9.793015	9.748945	21.373767
Si	13.138098	21.053855	12.683502	Si	8.178413	8.264902	8.340290
Si	11.959726	22.433960	14.245874	Si	9.669016	12.679874	12.590277
Si	14.423979	8.325033	2.929530	Si	13.951501	5.499438	17.050419
Si	15.890201	9.771037	4.166468	Si	14.177222	8.590521	8.278308
Si	14.573159	11.057556	5.718065	Si	6.663196	15.664527	12.632198
Si	15.971352	12.510213	7.051070	Si	6.825902	9.653207	18.642759
Si	14.627008	14.055057	8.369511	Si	8.246684	5.530167	5.527917
Si	15.931864	15.403713	9.914818	Si	18.584996	6.731922	9.809113
Si	14.528240	16.810431	11.338253	Si	5.180316	11.096210	2.600633
Si	15.990290	18.205251	12.720000	Si	19.807069	5.487371	11.466996
Si	14.621503	19.710510	14.013563	Si	21.193134	6.992158	12.756318
Sn	16.007471	21.265861	15.591242	Si	21.098194	9.909241	15.591846
Sn	17.437793	8.253922	5.601372	Si	21.070882	12.611429	18.487642
Si	18.871374	9.753784	7.184179	Si	12.666650	9.571696	1.144669
Si	17.388461	11.148163	8.488595	Si	8.398867	22.640772	11.462786
Si	18.720318	12.608434	9.884837	Si	6.972108	18.567547	15.663848
Si	17.361958	14.017126	11.336245	Si	3.767145	18.673270	12.720726
Si	18.760007	15.408066	12.755471	Si	9.923239	15.681991	21.570666
Si	17.335060	16.790066	14.177235	Si	21.153200	18.382433	12.681113
Si	18.701045	18.178940	15.627022	Si	22.502382	14.350913	11.504232
Si	17.342507	19.552233	17.060658	Si	5.056648	14.120558	5.475184

$Si_{222}Sn_{46}$(17% Sn-random)				$Si_{222}Sn_{46}$(17% Sn-core)			
Si	20.201184	8.298202	8.561463	Si	11.401484	22.504398	14.372635
Si	21.812456	9.558846	9.805707	Si	5.227467	17.235513	8.377100
Sn	20.280474	11.072508	11.314646	Si	5.315647	20.123807	11.564641
Si	21.700348	12.697267	12.795060	Si	9.896220	21.133581	15.620505
Si	20.160621	13.969472	14.123525	Si	11.074439	5.287380	2.697967
Sn	21.708704	15.438758	15.641350	Si	22.624723	11.240607	8.495148
Si	20.091482	16.879294	17.080972	Si	14.136293	11.306153	22.697598
Si	23.005996	8.271701	11.428813	Si	19.851561	19.741437	14.183297
Si	23.170046	11.438686	14.188533	Si	21.088405	15.483981	9.929519
Sn	12.976938	15.448807	1.159201	Si	2.365120	17.115821	11.503170
Si	11.663239	16.847550	2.912112	Si	18.468055	9.732293	12.827461
Si	13.194583	18.152058	4.175600	Si	21.288700	12.651665	7.073341
Sn	11.678132	19.732481	5.563900	Si	16.762016	16.888920	19.834754
Si	13.114766	21.161115	7.171135	Si	6.987542	21.285937	12.865591
Si	11.733010	22.459484	8.604418	Si	14.167511	8.451138	2.618788
Si	15.874067	12.482072	1.334144	Si	11.204815	2.599475	5.447610
Si	14.645044	14.073366	2.628409	Si	18.368090	12.802583	15.693654
Si	15.897168	15.333243	4.250145	Si	5.400780	8.340527	5.608778
Si	14.491871	16.750144	5.627873	Si	17.152575	5.334553	8.462074
Si	15.824101	18.149314	7.083764	Si	18.232800	15.525570	18.480110
Si	14.433197	19.590184	8.416788	Si	6.867239	4.105005	12.767111
Sn	15.982375	21.122649	9.855880	Si	19.837500	8.344367	14.225063
Si	14.610981	22.546378	11.545966	Si	15.490053	4.137246	15.737584
Si	17.250781	11.148959	2.749535	Si	6.908549	9.849801	12.891205
Si	18.666053	12.526572	4.104104	Si	11.256427	14.014448	11.373207
Si	17.288847	13.857593	5.541593	Si	16.865912	19.892079	17.028701

$Si_{222}Sn_{46}$(17% Sn-random)				$Si_{222}Sn_{46}$(17% Sn-core)			
Sn	18.813641	15.421615	6.976323	Si	9.715223	4.077611	4.248953
Si	17.273836	16.866366	8.527825	Si	9.785478	18.464279	18.444816
Si	18.722428	18.166418	9.927288	Si	14.226736	2.512270	14.469255
Si	17.442695	19.577397	11.354264	Si	8.431679	14.280871	2.615492
Si	18.889547	21.064674	12.529942	Si	14.035080	19.774240	19.731873
Sn	20.363756	11.195088	5.586840	Si	12.606602	18.662846	21.306581
Si	21.779209	12.598477	7.263703	Si	6.862477	7.001362	4.202020
Si	20.249635	13.916839	8.557114	Si	16.875440	11.324512	2.794780
Si	21.514592	15.368068	9.972306	Si	18.544628	15.432070	6.892775
Si	20.115347	16.780823	11.298542	Si	18.365666	12.692545	4.106561
Si	21.632733	18.218784	12.455816	Si	16.975757	2.546489	11.394416
Sn	20.317826	19.661739	14.187650	Si	15.470272	15.475879	21.305909
Si	23.134974	11.167433	8.620491	Si	14.124714	2.535273	8.376606
Si	22.959642	14.251844	11.496124	Si	15.423096	21.175509	15.577171
H	0.752723	8.189975	11.773441	H	7.527892	17.679573	3.958061
H	0.000000	10.381045	13.542435	H	10.246379	13.381706	0.047919
H	1.096501	11.600888	14.652048	H	21.794981	11.667525	19.486002
H	0.774057	13.287033	16.380091	H	22.143674	13.349908	17.682134
H	3.852357	2.512720	9.067372	H	15.213193	12.263142	23.284796
H	1.512601	5.603846	10.330784	H	20.729837	15.105682	20.510428
H	2.024177	3.812482	12.541767	H	8.399021	11.743584	0.635623
H	2.117361	7.179779	15.265181	H	7.564748	23.710759	10.701075
H	2.056678	10.493319	18.080717	H	15.150878	7.514756	1.818517
H	3.712588	13.404862	19.452758	H	22.120046	6.089427	13.660392
H	5.266064	1.738677	12.593060	H	7.377431	21.334806	7.822196
H	6.934345	1.443211	10.776612	H	9.318943	23.413687	12.429617

$Si_{222}Sn_{46}$(17% Sn-random)				$Si_{222}Sn_{46}$(17% Sn-core)			
H	5.171756	4.185860	15.428889	H	11.689662	8.502939	0.574901
H	5.415449	7.309679	18.116157	H	13.397599	10.282918	0.000000
H	5.076254	10.062508	20.555401	H	19.655688	11.966543	22.233742
H	6.362387	13.279143	22.304234	H	12.451088	23.086878	15.362791
H	10.907281	0.175214	13.120593	H	10.624761	23.621385	13.663995
H	9.119396	0.810076	11.436025	H	7.691011	15.371561	1.748671
H	7.983122	1.775873	14.931326	H	9.064073	7.361300	1.857704
H	7.857325	4.349621	17.691764	H	16.254182	22.069706	14.641867
H	7.843976	7.635243	20.940945	H	4.583616	21.208712	10.684336
H	9.789882	12.188750	23.427512	H	20.800140	4.519463	10.709027
H	8.343995	10.225886	23.851092	H	14.542166	22.079613	16.454864
H	14.028812	0.000000	16.016803	H	17.672900	17.790552	20.757479
H	12.339992	0.200396	14.215705	H	4.122331	11.842096	1.742150
H	10.340797	1.120704	17.640506	H	17.775902	7.638267	4.200367
H	14.366908	2.768152	19.497663	H	3.945071	14.848348	4.625191
H	10.689638	4.300032	20.973966	H	4.284274	18.211819	7.580896
H	14.217272	6.057663	22.729430	H	22.018339	9.108908	16.594314
H	12.473011	9.562898	23.600803	H	13.206743	22.187647	17.612155
H	10.620371	7.962536	23.956763	H	7.479689	9.279812	1.947287
H	1.025069	11.693995	8.520389	H	9.063517	22.020655	16.624857
H	0.458555	13.293050	10.406392	H	11.970975	4.286489	1.921502
H	0.969222	14.287204	11.867049	H	10.254899	6.090934	1.682654
H	0.641443	16.176399	13.415065	H	11.510622	21.761381	19.388193
H	3.499341	5.842875	5.989259	H	15.049059	4.456800	4.633441
H	1.944371	9.368637	7.468022	H	14.939628	20.755196	20.571795
H	1.897736	7.390830	9.552201	H	18.020737	4.550774	7.401395

$Si_{222}Sn_{46}$(17% Sn-random)				$Si_{222}Sn_{46}$(17% Sn-core)			
H	3.841002	16.362242	16.496717	H	17.725020	10.377501	1.862556
H	6.999929	2.862996	5.992527	H	14.450556	11.658534	0.733873
H	6.303677	16.290695	19.427858	H	19.405448	21.899398	11.819693
H	8.123079	1.839650	9.487424	H	23.839153	10.678353	7.696976
H	10.059197	1.676422	7.962211	H	17.677001	22.127693	13.592469
H	9.622815	16.480920	22.273212	H	13.437489	23.633475	10.826164
H	13.577594	0.254556	10.544538	H	15.041548	23.188577	12.741108
H	11.818024	0.796554	8.787160	H	19.210794	13.528546	3.063877
H	13.015258	14.957605	23.308059	H	19.548345	16.297634	6.036394
H	11.134801	13.327249	23.771183	H	19.351710	19.426649	9.319566
H	16.574813	0.125585	13.534677	H	20.826491	10.601514	4.552281
H	14.945009	0.462749	11.698815	H	23.602346	10.627201	13.649147
H	16.786095	3.140425	16.353384	H	16.213574	19.334973	5.711730
H	17.044052	5.756677	19.457249	H	22.316330	13.572985	6.304905
H	16.743755	8.547676	22.694796	H	22.066901	16.362354	9.056116
H	15.466338	12.581526	23.330766	H	22.200457	17.521050	13.390035
H	13.694211	10.876006	23.940842	H	21.910156	19.389926	11.774522
H	3.297460	8.732142	2.806346	H	20.875044	20.738609	14.854693
H	2.187098	12.444628	4.507587	H	6.051133	10.255168	1.661143
H	1.861606	10.596628	6.147762	H	16.590403	22.442684	9.422376
H	2.323597	15.181293	7.619857	H	16.284562	16.538466	3.153199
H	2.095374	17.426725	10.255982	H	20.907229	7.301751	7.536703
H	3.435112	19.407621	13.759429	H	12.295196	23.389876	9.445563
H	6.852233	5.793552	3.226817	H	11.630104	14.467464	0.756432
H	6.078279	18.899176	16.517474	H	21.975012	14.687069	16.535724
H	9.367440	3.318821	3.230635	H	21.954936	16.490128	14.811675

$Si_{222}Sn_{46}$(17% Sn-random)				$Si_{222}Sn_{46}$(17% Sn-core)			
H	9.388590	19.435242	19.971134	H	20.695992	17.552163	18.186601
H	10.975489	1.259089	6.358590	H	23.289425	9.373287	12.397686
H	13.109305	1.764487	4.492792	H	23.834435	7.520607	10.855564
H	12.083401	19.210920	21.896768	H	23.199737	12.275918	15.544272
H	13.896006	17.563822	22.411474	H	13.350459	16.157444	0.266379
H	15.321457	1.811358	7.633935	H	17.531496	20.949308	17.998033
H	15.040037	16.227075	22.218632	H	16.143932	13.381366	0.271719
H	16.715540	14.395488	22.195674	H	10.371917	17.785863	1.854784
H	18.207786	1.819333	10.385878	H	13.732085	19.040787	3.176433
H	19.554484	3.212684	13.709031	H	10.768657	20.945276	4.145701
H	19.514721	5.906600	16.434467	H	13.350332	22.643386	6.147240
H	20.100044	8.483779	19.234436	H	10.382439	23.908918	7.976539
H	18.193746	13.445441	22.710799	H	6.301790	22.289869	13.873744
H	20.386461	11.600796	22.327645	H	7.623572	4.525507	18.248758
H	6.921475	10.539464	1.548108	H	23.237042	12.218542	9.522445
H	5.143180	12.284836	1.482300	H	0.000000	16.011384	13.618225
H	5.184372	15.232444	4.864895	H	13.487720	3.218130	19.328266
H	5.072634	17.547308	7.538759	H	4.602293	10.133936	20.886807
H	5.125225	20.577209	10.197713	H	7.603075	7.422882	20.960179
H	6.337986	22.356639	13.160952	H	9.379255	12.269098	23.340239
H	9.931899	7.635632	1.928953	H	7.830592	10.446902	23.908966
H	8.026143	9.212724	2.081807	H	13.098985	0.000000	16.477533
H	9.680748	22.473954	16.565434	H	10.373698	1.714460	17.966498
H	12.853168	4.553580	2.097839	H	8.131892	0.421922	12.064959
H	11.159456	6.340633	1.794215	H	7.184391	1.982571	15.134016
H	13.950494	22.238003	18.065432	H	13.464025	6.044903	22.252302

	$Si_{222}Sn_{46}$(17% Sn-random)				$Si_{222}Sn_{46}$(17% Sn-core)		
H	12.231172	21.733622	19.807387	H	12.199099	9.303885	23.440213
H	15.300763	4.539719	4.852387	H	10.453705	7.607124	23.856041
H	15.509146	20.422365	20.801759	H	0.488681	11.322136	8.968515
H	18.430571	4.337424	7.494909	H	0.006695	13.322068	10.444511
H	18.269826	17.516351	20.845575	H	0.508623	14.571822	11.681613
H	20.991507	4.506791	10.441168	H	4.355305	7.466353	18.249198
H	22.478417	6.041931	13.706413	H	11.641901	0.638692	14.598275
H	22.797019	8.694176	16.579568	H	10.295991	4.574541	20.744203
H	22.060841	11.507532	19.649046	H	17.807316	13.647204	22.437870
H	22.892275	13.149498	17.947244	H	5.711334	13.038020	22.640992
H	21.233397	15.159732	20.545285	H	3.269069	3.147139	9.048046
H	8.847063	11.467614	1.059827	H	1.833423	4.295138	12.136738
H	10.781950	12.849104	0.213355	H	23.165943	15.497459	12.323592
H	8.255080	15.005592	1.807819	H	0.607206	8.833984	11.443346
H	7.998611	17.746212	4.919453	H	0.103697	10.311859	13.423098
H	8.111251	20.479910	7.514893	H	0.520673	11.583296	14.666902
H	8.442953	23.659331	10.341859	H	0.007559	13.511333	16.120877
H	9.966786	23.175798	12.213774	H	1.724120	6.182878	10.535108
H	11.922590	8.374747	0.913823	H	19.747579	9.148691	19.364875
H	13.623948	10.230135	0.440076	H	1.329619	7.413155	14.877115
H	11.227725	23.510923	13.437774	H	1.358707	10.496252	17.940048
H	13.079742	23.093287	15.099913	H	2.975030	13.195869	19.892405
H	15.323092	7.405994	2.016122	H	4.458117	1.988634	12.334097
H	17.093889	22.302293	14.682068	H	10.042501	0.069089	13.737852
H	14.874895	22.242029	16.515256	H	6.308663	1.645351	10.703883
H	18.498961	7.188053	4.645515	H	4.546438	4.197018	14.969430

Si$_{222}$Sn$_{46}$(17% Sn-random)				Si$_{222}$Sn$_{46}$(17% Sn-core)			
H	18.362696	20.354557	17.957009	H	3.024635	8.819574	3.413376
H	20.876791	7.300538	7.538746	H	3.303070	5.886953	6.123595
H	22.740902	14.368713	16.583824	H	13.428037	0.042902	10.349478
H	22.721145	16.414051	14.586111	H	10.404559	1.662619	6.359027
H	20.908359	17.897597	17.965673	H	1.582622	7.250233	9.175098
H	23.660452	9.286258	12.390539	H	11.886779	1.712148	4.367712
H	24.194897	7.465688	10.829303	H	1.292005	17.876235	10.633336
H	23.834005	12.376566	15.238377	H	2.838339	19.516286	13.678506
H	24.245201	10.761425	13.328719	H	5.909080	6.171363	3.255507
H	11.969424	14.305124	0.270177	H	6.111712	19.602374	16.486302
H	13.803414	16.500375	0.000000	H	8.792326	3.100404	3.421244
H	10.664286	17.818754	2.174400	H	19.285479	3.080612	13.553760
H	14.154581	18.964862	3.223017	H	1.452292	14.615582	7.382375
H	10.549631	20.633796	4.519932	H	11.966921	19.733952	22.234648
H	14.131365	22.068387	6.379169	H	2.591852	16.438602	16.358289
H	10.934706	23.541887	7.823190	H	13.402753	17.705112	22.201979
H	12.589064	23.231754	9.628264	H	14.922008	1.521063	7.460003
H	14.837846	11.554184	0.668568	H	14.486488	16.312282	22.145480
H	16.669595	13.134344	0.165441	H	16.450572	14.750730	22.246473
H	16.906710	16.327372	3.549837	H	17.965182	1.457943	10.812295
H	16.755117	19.042376	6.172430	H	8.953353	19.625084	19.121080
H	17.012136	22.139116	8.816287	H	1.537834	8.990930	7.334668
H	13.832740	23.676529	10.862515	H	19.564496	5.907427	16.369282
H	15.578680	23.132651	12.615293	H	1.905549	12.186322	4.581607
H	18.126487	10.153170	1.894225	H	13.366545	10.633858	23.837662
H	19.470020	13.521535	3.181814	H	5.994617	3.269931	6.107180

Si$_{222}$Sn$_{46}$(17% Sn-random)				Si$_{222}$Sn$_{46}$(17% Sn-core)			
H	19.808657	16.461944	5.922724	H	6.008610	16.926057	19.406786
H	19.679674	19.055100	9.042547	H	7.262836	1.807503	9.200312
H	19.818297	21.695280	11.458649	H	9.163434	1.921401	7.579435
H	18.067251	22.201091	13.157693	H	9.178845	16.420492	22.750314
H	21.375963	10.114610	4.591498	H	11.772407	0.429705	8.468182
H	22.713952	13.626119	6.515888	H	1.623941	10.436037	6.323329
H	22.361881	16.311921	9.036683	H	12.611962	15.088944	23.286346
H	22.785310	17.359307	12.996126	H	10.705969	13.465630	23.706996
H	22.222870	19.194648	11.404465	H	16.183038	0.064133	13.330739
H	21.378992	20.642846	15.231062	H	14.377120	0.705646	11.816932
H	23.841801	12.047108	9.670256	H	16.344508	3.398403	16.847267
H	24.272350	10.527360	7.771564	H	15.919728	5.696753	19.562793
H	23.580147	15.314129	12.443674	H	16.121251	8.748386	22.390893
H	24.048698	13.470949	10.748402	H	23.556168	13.556462	10.718224

Si$_{222}$Sn$_{46}$(17% Sn-shell)				β-Sn$_{268}$			
Atom	x [Å]	y [Å]	z [Å]	Atom	x [Å]	y [Å]	z [Å]
Sn	1.244070	9.900954	12.622158	Sn	11.224692	6.506807	3.937275
Sn	15.431581	1.143716	12.821944	Sn	13.809000	10.482173	1.301133
Sn	18.324857	15.752202	7.078017	Sn	5.336336	7.250049	6.573435
Sn	11.121895	17.230037	2.569443	Sn	8.586923	7.586643	3.965415
Sn	9.535311	1.553742	12.654558	Sn	8.265797	10.097106	5.391347
Sn	9.762469	21.427577	15.729780	Sn	11.531275	10.217909	3.071711
Sn	19.787451	11.493601	5.643103	Sn	10.912958	12.995199	3.575118

$Si_{222}Sn_{46}$(17% Sn-shell)				β-Sn_{268}			
Sn	8.530044	8.774367	2.621456	Sn	13.799456	15.499244	1.305985
Sn	21.226421	15.797489	15.685647	Sn	8.479681	4.107005	8.351866
Sn	22.734406	8.740972	11.386871	Sn	10.845480	7.393352	7.460892
Sn	22.788418	11.789052	14.475872	Sn	13.914878	7.530914	4.138343
Sn	12.729119	18.753797	4.280486	Sn	14.290706	10.043585	5.704130
Sn	15.508951	4.361652	15.686558	Sn	16.437394	10.588888	2.407401
Sn	14.324983	22.853643	11.601690	Sn	16.709364	12.995019	3.986894
Sn	12.541489	1.597923	9.706325	Sn	14.207567	4.110283	8.469119
Sn	6.886577	7.194560	4.244298	Sn	16.884116	7.097921	6.828078
Sn	4.120444	18.669642	12.817976	Sn	19.533842	10.246060	5.053851
Sn	22.838185	14.515850	11.661003	Sn	2.561644	10.219264	8.116802
Sn	2.624403	8.687699	8.646131	Sn	5.396164	10.052694	5.914174
Sn	9.854642	4.369280	4.128979	Sn	5.439631	12.999038	6.825080
Sn	8.389299	2.896487	8.580526	Sn	8.106684	12.989379	4.653662
Sn	12.689892	21.688027	7.267367	Sn	8.271324	15.893094	5.348573
Sn	12.751417	15.554639	1.035258	Sn	11.517665	15.777603	3.079172
Sn	12.776410	7.174566	21.463857	Sn	11.228392	19.472514	3.875502
Sn	11.635179	2.755010	5.561435	Sn	4.699493	7.220191	10.404510
Sn	21.232186	15.937223	9.884267	Sn	7.786184	6.899811	8.147381
Sn	4.120686	4.374617	10.043828	Sn	8.229839	10.032930	8.489854
Sn	21.199079	9.922115	15.802997	Sn	11.232191	10.025160	6.058334
Sn	5.549910	8.666892	17.115028	Sn	11.175900	12.998603	6.858153
Sn	8.435668	8.719688	20.039894	Sn	13.799057	12.995835	4.709310
Sn	3.861281	10.022697	4.215436	Sn	14.296057	15.940753	5.666153
Sn	14.343653	11.789470	22.770149	Sn	16.437983	15.395702	2.398154
Sn	2.597765	11.568507	17.149758	Sn	8.007974	3.274370	12.160254

$Si_{222}Sn_{46}$(17% Sn-shell)				β-Sn_{268}			
Sn	12.544256	1.284432	15.655770	Sn	11.261044	4.403935	9.038443
Sn	5.404684	11.764641	2.619036	Sn	11.222858	6.891370	10.753393
Sn	1.148100	15.719774	12.795890	Sn	14.022644	7.088528	7.884784
Sn	11.301485	8.782378	23.218876	Sn	13.994344	10.107876	8.829371
Sn	12.505499	18.706773	21.379675	Sn	17.134408	10.024900	6.734305
Sn	19.750792	14.393073	19.973645	Sn	16.927566	13.011777	7.009776
Sn	17.023483	2.750822	11.171878	Sn	20.195153	12.994244	4.558228
Sn	12.582241	4.303875	18.581635	Sn	19.535245	15.748217	5.018561
Sn	18.569943	4.389077	12.819968	Sn	11.272005	1.321489	13.699883
Sn	8.411792	17.261327	5.749357	Sn	14.081135	4.488865	12.356681
Sn	8.394605	11.746678	22.937199	Sn	17.099290	4.601779	8.936498
Sn	5.520117	14.359004	5.754084	Sn	17.394871	7.060042	10.610034
Sn	2.292767	11.823935	5.706317	Sn	19.708105	7.265212	7.568478
Si	18.274693	12.732837	21.320439	Sn	19.906834	9.895224	8.963529
Si	16.866831	8.725157	16.995612	Sn	19.972000	4.239367	11.670414
Si	16.923343	5.919179	14.162402	Sn	2.473979	12.992052	7.227740
Si	7.084567	18.578409	10.059766	Sn	2.583874	15.803470	8.031622
Si	18.201667	10.164947	18.387557	Sn	5.410719	15.933669	5.888073
Si	11.222574	11.524382	8.595996	Sn	5.352035	18.732841	6.569649
Si	9.813142	12.930011	7.176152	Sn	8.571286	18.428329	3.957438
Si	18.301305	12.923258	10.018652	Sn	2.506041	10.537964	11.912639
Si	7.049645	4.516653	12.814659	Sn	5.215635	9.958298	9.381196
Si	16.863451	8.734714	11.424963	Sn	5.408497	12.995404	10.044779
Si	15.418163	15.710311	21.149508	Sn	8.292074	12.999511	8.103177
Si	18.230744	7.351555	15.552451	Sn	8.193257	15.962083	8.427589
Si	7.002236	15.774863	7.340926	Sn	11.229069	15.976119	6.069827

$Si_{222}Sn_{46}$(17% Sn-shell)				β-Sn_{268}			
Si	14.057209	14.325092	19.760298	Sn	10.860666	18.650394	7.420815
Si	8.371431	19.883642	14.163376	Sn	13.927147	18.470626	4.127887
Si	8.412342	11.492653	5.784776	Sn	5.261568	4.056233	12.502198
Si	12.682817	7.327174	4.441273	Sn	5.134239	6.852849	13.326846
Si	5.727516	20.073847	11.352038	Sn	8.234027	7.034799	11.172596
Si	11.588891	5.872761	2.892962	Sn	8.143041	9.948142	11.856861
Si	9.858002	18.530816	12.824027	Sn	11.086982	9.857891	9.684606
Si	8.453153	17.133352	11.433665	Sn	11.172455	13.005560	10.207291
Si	9.847873	15.742406	10.009634	Sn	14.072343	13.008028	7.967468
Si	18.290642	10.131903	12.812602	Sn	13.932172	15.918396	8.773030
Si	18.298258	21.332259	12.732736	Sn	17.133793	16.004002	6.693288
Si	7.069535	21.372953	12.815183	Sn	16.887492	18.930529	6.817083
Si	12.643084	12.917941	10.003710	Sn	8.148380	4.096698	15.806047
Si	8.503956	14.234600	2.988168	Sn	10.899748	3.966670	12.562187
Si	18.287045	12.939134	15.617752	Sn	11.118480	7.020705	13.842455
Si	19.664253	8.649156	14.137608	Sn	14.326873	7.163280	10.979585
Si	1.516232	12.784902	9.839790	Sn	14.197814	9.934458	12.226390
Si	16.871048	14.333115	17.012504	Sn	16.896151	10.056966	9.898289
Si	5.613715	11.494374	8.621824	Sn	17.200797	13.012049	10.664441
Si	5.714126	5.930076	14.198133	Sn	19.650164	12.985823	8.280352
Si	14.035691	5.967655	5.864588	Sn	19.882048	16.080872	8.917446
Si	16.878194	11.521426	14.228880	Sn	13.982968	1.308338	14.557429
Si	18.199766	15.803534	18.404461	Sn	14.409217	3.927690	15.735524
Si	16.759078	17.133744	19.786329	Sn	17.133692	4.025676	12.445601
Si	19.833950	6.016540	11.269645	Sn	17.221681	6.893588	13.794529
Si	14.068054	19.938096	8.643832	Sn	20.265338	7.121744	11.448442

$Si_{222}Sn_{46}$(17% Sn-shell)				β-Sn_{268}			
Si	15.369820	15.669979	4.371931	Sn	20.045436	10.031516	12.164441
Si	16.856471	11.526851	19.815088	Sn	22.768871	10.243519	9.039067
Si	11.241713	14.339853	5.777466	Sn	22.937426	12.993556	9.908851
Si	21.110975	12.691575	18.563256	Sn	20.014863	4.110466	15.668456
Si	9.719071	12.685056	1.652583	Sn	22.867065	7.062394	13.914558
Si	11.230886	8.686240	5.811540	Sn	1.998940	13.062959	10.413356
Si	9.958807	18.687664	7.277198	Sn	2.380060	15.623669	11.837751
Si	15.454129	7.333757	7.232239	Sn	5.218999	16.035944	9.381066
Si	8.522902	19.965190	8.707287	Sn	4.748370	18.815998	10.356732
Si	9.842172	21.408832	10.112844	Sn	7.816572	19.088791	8.128556
Si	8.531584	22.766896	11.572414	Sn	8.458536	21.893843	8.355718
Si	12.496804	9.956000	1.724664	Sn	2.376634	7.001008	14.268405
Si	11.263160	11.513989	3.056561	Sn	2.213696	9.619457	15.514300
Si	12.661670	12.943801	4.393643	Sn	5.270870	10.100180	12.811686
Si	9.886846	15.680988	4.284172	Sn	5.063828	12.999471	13.662114
Si	5.636789	8.688755	11.434458	Sn	8.294366	13.022102	11.348755
Si	9.846743	10.096368	15.658694	Sn	8.076036	16.071102	11.852130
Si	5.646180	5.924703	8.578277	Sn	11.027314	16.162345	9.684793
Si	4.241883	7.282719	7.189461	Sn	11.254960	19.119779	10.710824
Si	7.032168	7.301175	10.010284	Sn	14.031399	18.944048	7.882810
Si	15.414803	18.558807	18.403585	Sn	14.212999	21.916088	8.480635
Si	11.289474	19.847693	17.109087	Sn	5.296068	4.317155	16.527614
Si	12.560050	21.281750	18.529080	Sn	5.033010	7.185142	16.827556
Si	5.659282	17.153728	8.746727	Sn	7.998767	6.941708	14.401846
Si	14.057225	11.509628	17.026727	Sn	8.220065	9.936897	15.055393
Si	14.046117	8.718730	8.625880	Sn	11.127565	10.088503	12.849290

$Si_{222}Sn_{46}$(17% Sn-shell)				β-Sn_{268}			
Si	14.047667	11.518084	11.421240	Sn	11.150526	13.022556	13.596012
Si	15.475020	12.919895	12.811200	Sn	14.077346	12.998937	11.436394
Si	14.058382	14.315953	14.218976	Sn	14.222342	16.075788	12.185045
Si	14.042504	17.127206	17.016798	Sn	16.856359	15.973092	9.874357
Si	15.449906	15.723540	15.628611	Sn	17.422566	18.955918	10.589806
Si	4.209829	12.941165	12.850736	Sn	19.716404	18.735128	7.556115
Si	15.455731	10.128089	10.014933	Sn	8.125601	4.518680	19.289591
Si	18.279461	7.358008	10.029868	Sn	11.226748	4.465169	15.925896
Si	15.472696	12.921600	18.404150	Sn	10.980698	7.121645	17.362548
Si	14.060148	8.687903	14.213356	Sn	14.177072	6.967777	14.513599
Si	11.244013	8.691288	11.403626	Sn	14.149043	10.058360	15.502348
Si	6.990879	4.539682	7.156483	Sn	17.101459	9.929205	13.233755
Si	4.255179	15.661871	15.612767	Sn	17.259186	12.994595	13.720272
Si	16.851874	5.989044	8.659391	Sn	20.305184	13.002274	11.426924
Si	14.110478	20.008658	19.800919	Sn	19.949491	15.975316	12.116656
Si	2.796246	14.313064	14.257954	Sn	22.757612	15.772672	9.118347
Si	5.561913	3.002097	11.716770	Sn	13.990126	4.379138	19.283125
Si	7.039737	10.085298	12.838322	Sn	17.296757	3.233089	16.062896
Si	11.241755	14.321052	11.412389	Sn	17.116472	7.044455	17.098685
Si	8.444269	11.523738	17.043368	Sn	20.085258	6.922892	14.866123
Si	7.079706	12.860458	4.308018	Sn	20.099073	10.068429	15.543366
Si	11.234651	17.107823	14.230045	Sn	23.172414	9.819200	12.984131
Si	8.434195	14.318041	19.805284	Sn	22.936507	13.054045	13.465245
Si	5.613029	11.503298	14.217129	Sn	2.490445	13.351444	15.156766
Si	4.248194	13.042798	18.508838	Sn	1.824906	16.205737	15.448151
Si	7.086018	15.689264	18.361266	Sn	5.165614	15.855124	12.780714

$Si_{222}Sn_{46}$(17% Sn-shell)				β-Sn_{268}			
Si	9.875074	4.496169	10.037916	Sn	5.221050	19.112268	13.296459
Si	8.446950	5.894399	11.400059	Sn	8.241074	18.988213	11.173942
Si	9.860608	7.277346	12.821832	Sn	7.998888	22.761097	12.163794
Si	8.469943	8.692188	14.235470	Sn	11.255884	21.613508	9.017614
Si	5.647126	14.303852	17.022068	Sn	2.515250	10.347342	19.213509
Si	7.128358	7.236397	15.615720	Sn	5.166041	10.065130	16.061087
Si	7.028750	12.894594	15.622275	Sn	5.417519	13.009417	17.068106
Si	2.843553	8.645958	14.228125	Sn	8.015291	13.027197	14.665528
Si	4.269782	7.299190	12.865534	Sn	8.319385	16.146721	14.958265
Si	1.226668	13.046989	15.536230	Sn	11.167369	15.949862	12.824550
Si	2.772387	5.813605	11.739617	Sn	11.145193	19.022910	13.806664
Si	4.257475	10.140045	10.087363	Sn	14.345154	18.861994	10.977594
Si	2.849606	11.571089	11.408043	Sn	14.081917	21.528062	12.371456
Si	7.009121	10.284514	18.532358	Sn	17.111344	21.410387	8.926398
Si	9.830536	15.706185	21.161707	Sn	5.514713	7.305876	20.581483
Si	4.112896	10.159197	15.552772	Sn	7.919800	7.024373	17.657738
Si	15.452484	10.110232	15.608146	Sn	8.399096	10.036944	18.450672
Si	12.635479	18.501979	15.627784	Sn	11.107554	9.875000	16.108598
Si	21.257347	12.927571	7.228967	Sn	11.194057	12.991408	16.697232
Si	9.856855	12.903598	18.451490	Sn	14.223058	12.996272	14.642543
Si	16.843013	19.921697	11.464239	Sn	14.158668	15.942708	15.464058
Si	12.663093	4.536710	7.193829	Sn	17.088882	16.074548	13.251181
Si	8.475110	5.837524	5.806183	Sn	17.245085	19.098314	13.779268
Si	4.208884	15.768344	10.075871	Sn	20.299485	18.862096	11.423170
Si	5.516368	8.763431	5.814803	Sn	20.001181	21.749527	11.656050
Si	12.651378	15.721258	18.397284	Sn	10.992711	4.054853	19.809826

$Si_{222}Sn_{46}$(17% Sn-shell)				β-Sn_{268}			
Si	6.964484	13.102576	21.253848	Sn	11.213869	7.023503	20.445507
Si	12.652371	12.906047	15.619115	Sn	14.117793	6.866125	17.584800
Si	11.286455	5.856194	14.218526	Sn	14.142772	9.833637	18.615850
Si	5.628667	11.685919	19.888578	Sn	17.189972	9.949068	16.421263
Si	5.609737	14.335141	11.438502	Sn	17.050635	13.001941	16.989748
Si	8.355659	3.175800	14.294376	Sn	20.126359	12.990230	14.693998
Si	9.886558	4.451797	15.613082	Sn	20.100658	15.903413	15.513248
Si	12.656857	10.130296	18.471651	Sn	23.288248	16.138149	13.074068
Si	21.103294	12.913251	12.805617	Sn	22.787837	18.912051	13.896950
Si	9.977663	10.246726	21.497080	Sn	16.807281	4.160799	19.893101
Si	18.186913	18.514427	10.097437	Sn	17.408329	6.958441	20.183166
Si	19.614321	8.771849	8.629452	Sn	20.316841	7.109023	17.975912
Si	8.449263	17.113149	16.992833	Sn	20.121526	9.979746	18.964532
Si	12.685671	4.420088	12.828407	Sn	22.868556	10.071438	16.639389
Si	11.271225	17.166496	8.625438	Sn	23.080290	13.010242	17.337640
Si	11.240160	11.497268	14.229554	Sn	2.511168	18.819638	14.383811
Si	8.416089	11.501449	11.413714	Sn	5.260841	21.934472	12.524876
Si	9.837709	12.898703	12.809641	Sn	1.795126	12.839220	17.943139
Si	8.428904	14.296123	14.210868	Sn	2.565360	15.384776	19.098575
Si	9.833419	15.690203	15.614365	Sn	5.498935	15.999733	16.052804
Si	9.871212	18.436561	18.395777	Sn	5.013542	18.837866	16.806065
Si	4.100612	12.842552	7.304503	Sn	8.047052	19.169101	14.436538
Si	14.094780	8.750962	19.841465	Sn	8.130338	21.986758	15.782598
Si	11.248094	14.305028	17.024086	Sn	10.888226	22.056849	12.571893
Si	12.654435	7.295602	15.632164	Sn	11.260245	24.708904	13.696215
Si	16.819507	17.176308	8.662748	Sn	5.387387	9.984357	19.218929

$Si_{222}Sn_{46}$(17% Sn-shell)				β-Sn_{268}			
Si	18.266396	15.719396	12.839571	Sn	5.555952	12.987464	20.156642
Si	12.655271	13.013309	21.200387	Sn	8.335862	12.994039	17.743947
Si	2.678780	17.158585	11.286381	Sn	8.436820	15.962885	18.445635
Si	22.537307	11.532290	8.653463	Sn	11.123165	16.120479	16.144811
Si	2.877208	14.349527	8.677941	Sn	10.964976	18.916863	17.331043
Si	19.573508	19.987802	14.226441	Sn	14.185167	19.050215	14.521882
Si	9.824962	10.103833	9.998115	Sn	14.386612	22.099039	15.744850
Si	11.275061	8.707880	17.057720	Sn	17.151304	21.962843	12.430984
Si	15.470936	7.292243	12.802422	Sn	8.321518	7.124641	21.382031
Si	15.524796	10.105560	21.187715	Sn	8.037614	10.039695	21.600821
Si	9.827720	7.284426	7.199807	Sn	11.265946	10.057367	19.537134
Si	13.989489	2.872586	14.161614	Sn	11.362777	12.996049	20.252250
Si	11.222017	17.069156	19.796781	Sn	14.065756	12.990836	17.958818
Si	14.068691	5.859613	11.424516	Sn	14.149482	16.188161	18.589375
Si	15.493162	4.522408	9.990197	Sn	17.202986	16.057314	16.453985
Si	14.156532	3.230972	8.500848	Sn	17.042226	18.978324	17.080591
Si	11.265026	11.533418	19.899072	Sn	20.048287	19.114314	14.953796
Si	11.435728	14.593035	22.513026	Sn	19.989478	21.944257	15.679816
Si	6.996108	10.109506	7.187212	Sn	11.414328	7.494782	24.185363
Si	9.899872	7.266481	18.434313	Sn	14.376954	7.400646	20.843743
Si	8.555665	5.890125	16.993131	Sn	14.023204	10.024276	22.249076
Si	15.431324	7.380946	18.392329	Sn	17.014012	10.038183	19.690826
Si	16.850019	17.118865	14.228236	Sn	16.914807	13.004524	20.118395
Si	12.624668	10.100241	7.211915	Sn	20.035663	12.989665	18.073741
Si	14.004700	5.941659	17.108366	Sn	20.059317	16.026205	18.935171
Si	11.234546	5.889660	8.604067	Sn	22.832906	15.995286	16.715553

$Si_{222}Sn_{46}$(17% Sn-shell)				β-Sn_{268}			
Si	19.689169	14.292589	14.183693	Sn	16.681453	7.676337	24.331832
Si	14.067397	17.097932	5.810728	Sn	19.897781	7.215966	21.693388
Si	19.627497	11.556225	17.079276	Sn	19.887129	10.035527	22.196222
Si	18.224036	10.117941	7.222927	Sn	22.877923	10.371915	20.123570
Si	11.251788	19.963412	11.442889	Sn	22.599802	13.034336	21.243844
Si	20.931839	18.751012	12.712221	Sn	5.293506	21.716637	16.554791
Si	19.606856	17.195007	11.480127	Sn	5.418609	16.023130	19.218003
Si	19.774730	14.211356	8.589780	Sn	5.529119	18.712578	20.562877
Si	7.053502	18.455003	15.570264	Sn	7.904971	18.992194	17.670459
Si	12.629805	15.739999	7.200355	Sn	8.129134	21.533530	19.260001
Si	14.022826	11.520033	5.817165	Sn	11.197100	21.600739	15.952595
Si	15.549030	21.255123	9.978074	Sn	13.970770	24.718141	14.556940
Si	19.558562	17.244053	17.052946	Sn	5.410982	10.399799	22.956220
Si	12.643878	15.716418	12.823694	Sn	5.532228	12.988847	24.158773
Si	11.189573	20.146322	5.835609	Sn	8.485080	13.006522	21.295249
Si	11.139017	22.995019	8.877544	Sn	8.058709	15.972178	21.608333
Si	14.049193	14.278530	2.925225	Sn	11.302779	15.941420	19.579612
Si	15.397160	18.490514	7.258344	Sn	11.214225	18.987164	20.409328
Si	16.789974	11.454485	2.942856	Sn	14.047858	19.161165	17.609807
Si	9.870191	10.123796	4.431229	Sn	13.976885	21.649404	19.308833
Si	21.115802	7.295664	12.799641	Sn	17.268060	22.809235	16.061228
Si	19.713375	11.510685	11.402875	Sn	8.893271	10.578879	25.835990
Si	18.152285	12.918401	4.223779	Sn	10.936929	9.984492	22.624451
Si	12.658126	7.278306	10.013524	Sn	11.458987	12.993998	23.426239
Si	16.763930	14.227371	5.669493	Sn	14.170723	13.000279	21.519948
Si	12.663030	18.555570	10.050012	Sn	14.028279	15.984394	22.237561

$Si_{222}Sn_{46}$(17% Sn-shell)				β-Sn_{268}			
Si	15.388047	12.812202	1.596071	Sn	16.982103	15.967763	19.735115
Si	8.437830	14.327477	8.624873	Sn	17.395984	19.052824	20.161080
Si	14.034616	8.752269	3.086147	Sn	20.323299	18.929035	18.000160
Si	12.644696	10.095706	12.816886	Sn	14.093503	6.459878	24.343024
Si	8.414755	8.701304	8.586765	Sn	13.735680	10.269600	25.209924
Si	7.041316	15.718226	12.817079	Sn	17.026468	10.161443	22.844516
Si	7.009777	12.926589	10.016242	Sn	17.273853	13.008590	23.732324
Si	16.863218	11.541243	8.619673	Sn	19.669915	13.002576	21.343458
Si	16.798682	8.765197	5.855745	Sn	19.886670	15.983551	22.176230
Si	18.196313	18.581659	15.605863	Sn	22.902944	15.674046	20.052629
Si	11.090508	5.876692	19.974936	Sn	5.429234	15.584560	22.959703
Si	11.253072	3.093979	11.417170	Sn	8.332800	18.880799	21.344050
Si	21.078392	10.153758	9.944665	Sn	10.980247	21.966830	19.817355
Si	14.022008	19.885407	14.224390	Sn	8.389985	12.984384	24.374256
Si	11.509213	22.781590	14.381641	Sn	8.885198	15.380494	25.850963
Si	11.056231	2.906274	17.017401	Sn	10.938189	15.991018	22.657132
Si	15.440022	10.143182	4.430342	Sn	11.396987	18.496434	24.183502
Si	16.754353	19.943320	16.964305	Sn	14.374826	18.622273	20.847078
Si	12.643446	21.316174	12.876968	Sn	16.800596	21.852271	19.893800
Si	16.876382	14.335028	11.404817	Sn	11.486940	10.480920	26.982326
Si	14.050566	17.118952	11.429276	Sn	14.469122	13.003952	24.694661
Si	15.442160	18.510595	12.835914	Sn	13.732415	15.740651	25.199365
Si	5.629548	17.068475	14.238477	Sn	17.034153	15.863072	22.864085
Si	15.444141	15.722121	10.012404	Sn	16.667250	18.364546	24.324117
Si	14.040955	14.331043	8.597343	Sn	19.889393	18.802019	21.679842
Si	15.438511	12.911178	7.203950	Sn	11.485632	15.503787	26.981585

$Si_{222}Sn_{46}$(17% Sn-shell)				β-Sn_{268}			
Si	15.371128	21.311657	15.587275	Sn	14.064068	19.557652	24.355952
H	14.937611	7.827416	2.184021	H	11.243380	4.889641	3.186580
H	8.717787	11.626835	1.137466	H	13.698824	9.273111	0.002059
H	7.363575	18.285215	4.737199	H	13.531284	12.095603	0.588342
H	6.233090	11.084074	1.227590	H	3.954338	6.988556	7.688863
H	7.702143	9.916343	1.574132	H	5.232539	5.965316	5.343062
H	22.297365	13.273330	17.782517	H	7.774953	6.356928	4.994167
H	20.748349	15.450557	21.002304	H	7.844909	7.449697	2.358524
H	7.597535	20.844994	7.779071	H	10.145812	9.479367	2.160345
H	10.433002	13.305443	0.443332	H	13.527918	13.881317	0.599135
H	7.565752	15.125435	2.087345	H	13.686574	16.702565	0.000000
H	11.476575	8.948596	1.141822	H	7.818758	3.440810	9.925033
H	7.757979	23.811020	10.719999	H	8.199389	2.806139	7.155320
H	21.671715	11.591077	19.502215	H	11.163823	5.985335	5.716327
H	9.493516	23.574132	12.467124	H	14.545812	6.039900	4.893152
H	8.719656	22.405300	16.790308	H	14.611795	7.620878	2.497620
H	10.889058	23.943567	13.595095	H	16.648382	9.184136	3.480354
H	13.206632	10.692520	0.587400	H	17.664421	10.405661	1.128174
H	12.597107	23.301902	15.361620	H	14.158515	2.511885	7.656214
H	4.499073	15.390384	4.725434	H	16.833041	7.005160	5.019227
H	23.557725	10.625735	7.911109	H	21.026091	9.989636	6.008493
H	4.390817	13.125048	2.109500	H	19.695813	9.314295	3.549115
H	4.899084	20.957051	10.343147	H	1.153034	9.886349	9.178656
H	21.964854	8.808255	16.965197	H	2.312814	9.196222	6.655811
H	14.425836	22.220237	16.387678	H	4.479182	10.155213	4.353489
H	9.704500	7.891849	1.660040	H	7.160318	12.991216	3.107950

$Si_{222}Sn_{46}$(17% Sn-shell)				β-Sn_{268}			
H	16.228494	22.196556	14.669290	H	10.125849	16.497760	2.161418
H	20.749911	5.256826	10.234972	H	11.253538	21.100724	3.130339
H	17.728532	7.882088	4.936388	H	2.915756	7.067133	10.336262
H	6.163109	22.198273	13.805958	H	5.328014	5.779113	9.528587
H	14.913909	5.031394	4.949723	H	17.661747	15.590605	1.116065
H	17.760987	5.066839	7.756950	H	16.641425	16.805289	3.467618
H	12.692325	4.932871	2.341350	H	8.072619	1.490558	11.952992
H	17.689386	18.021654	20.698723	H	11.231827	3.276618	10.634327
H	11.054034	6.694314	1.713122	H	21.818895	12.985326	3.797781
H	21.845735	6.303095	13.784199	H	19.702335	16.700622	3.524486
H	13.177868	22.375616	17.653542	H	21.017627	16.018109	5.983249
H	11.542977	21.964351	19.480990	H	10.205963	1.262532	15.129887
H	15.090933	20.933083	20.619289	H	10.788553	0.013629	12.590309
H	16.537898	22.041800	9.037440	H	14.189644	3.647250	10.301339
H	13.807995	22.686116	6.304816	H	16.980519	3.375808	10.409239
H	4.767211	18.054139	7.806847	H	18.654499	4.038946	8.246600
H	20.471173	20.886615	15.160643	H	20.286902	5.725856	8.260683
H	15.511685	23.385618	12.793306	H	20.575631	7.481802	6.013228
H	19.263328	22.010515	11.725405	H	21.294714	3.363549	12.473159
H	16.332523	16.587778	3.521698	H	19.926259	3.738165	9.956200
H	19.182963	11.686136	22.018239	H	1.361715	12.972282	5.787345
H	17.666319	10.453226	2.095260	H	2.376443	16.751333	6.518048
H	18.935176	13.930168	3.303023	H	1.108329	16.150560	9.002653
H	19.117127	19.420906	9.204598	H	4.477400	15.832705	4.341648
H	23.787416	9.817541	12.306841	H	5.202010	20.044002	5.368867
H	20.723181	10.352416	4.643888	H	3.974604	18.965438	7.709038

$Si_{222}Sn_{46}$(17% Sn-shell)				β-Sn_{268}			
H	16.304407	19.401851	6.343300	H	7.784366	18.601496	2.372380
H	22.124567	13.970097	6.424205	H	7.802760	19.652452	5.028131
H	21.964327	17.079584	8.726956	H	1.971571	10.525794	13.766237
H	22.108554	18.029643	13.378075	H	1.752431	8.959132	11.481757
H	21.513991	19.728553	11.656481	H	11.171084	19.991414	5.670720
H	11.826129	14.412131	0.070656	H	14.676554	18.441810	2.508804
H	23.382483	12.360101	9.646800	H	14.482974	19.970981	4.928012
H	20.498710	7.836275	7.716886	H	4.596512	2.910502	13.700605
H	14.416590	11.968691	0.756652	H	4.362115	3.821752	10.987222
H	10.122388	20.916884	4.968073	H	16.818591	19.127269	5.021674
H	17.678109	20.841562	17.873199	H	8.056037	3.039891	14.131626
H	23.219093	13.101316	15.580621	H	20.722137	13.003316	6.374033
H	17.487359	22.432904	13.414421	H	14.415048	0.000000	15.687731
H	13.901486	16.410130	0.000000	H	15.038002	1.193091	13.123836
H	22.207011	14.785562	16.730002	H	17.202868	2.905225	14.100095
H	22.257134	16.769213	14.650291	H	21.523485	7.323365	10.140804
H	20.357290	18.190103	18.028190	H	23.462891	9.424824	10.480321
H	23.739821	7.729650	10.344868	H	23.911682	9.891201	7.701414
H	19.353797	16.795026	6.064635	H	24.151560	12.985627	11.286859
H	24.197685	11.226469	13.588355	H	20.959876	3.854519	17.161755
H	11.940962	23.897332	9.839359	H	20.683814	2.947602	14.475555
H	16.188078	13.666639	0.574011	H	24.003305	6.669089	15.235309
H	13.451252	24.181842	10.864900	H	23.089174	5.823412	12.626769
H	9.855616	18.206920	1.775094	H	0.185743	12.986769	10.547000
H	13.912632	19.813043	3.469675	H	1.969615	17.168873	11.035353
H	10.197998	23.936766	8.077737	H	1.342239	15.863634	13.362692

$Si_{222}Sn_{46}$(17% Sn-shell)				β-Sn_{268}			
H	13.716594	3.308751	19.527079	H	5.344357	20.301582	9.534111
H	12.991005	2.180443	4.580371	H	2.961829	18.936288	10.309809
H	9.364271	12.789630	23.971925	H	8.177557	23.210110	7.172550
H	7.374195	7.665151	21.008587	H	7.835458	22.558839	9.950891
H	19.137106	9.274051	19.291054	H	2.187424	5.706548	15.491566
H	13.659130	0.394860	16.698572	H	1.235091	6.716157	12.930617
H	10.319325	0.156367	13.373446	H	0.610259	10.249727	16.008534
H	9.978501	2.184887	17.912372	H	14.188593	23.507192	7.651835
H	7.643192	4.978056	17.901527	H	5.309229	3.836447	18.247457
H	1.592982	9.752181	7.701029	H	3.954874	3.474358	15.708476
H	10.093924	7.852523	24.116502	H	3.706240	7.301246	18.079603
H	16.514914	9.179526	21.991499	H	20.564488	18.541147	5.990392
H	7.421752	10.669239	23.946663	H	20.293002	20.276488	8.256338
H	11.656862	0.129121	14.668513	H	6.645228	3.949151	20.125544
H	0.110482	16.690254	13.844945	H	8.196608	3.294609	17.823143
H	8.372617	1.069073	11.421536	H	11.086272	3.624718	17.961222
H	7.350656	2.428991	15.252451	H	24.003137	16.240411	7.916666
H	13.932565	6.008605	22.164187	H	23.352726	16.470311	10.678773
H	12.233163	9.765479	24.346545	H	14.072536	3.273234	17.658908
H	1.059666	11.742936	8.784432	H	17.250394	1.465898	16.387845
H	10.014615	5.117107	20.842205	H	24.909196	9.921173	12.511814
H	14.486157	16.480867	22.100543	H	23.598864	13.034389	15.505952
H	23.472073	15.496195	12.989148	H	1.081229	12.960082	14.080711
H	4.531653	7.632676	18.146713	H	0.055325	16.282020	15.734700
H	0.411325	12.162111	14.571245	H	8.054329	24.540334	11.917078
H	2.190815	4.875756	12.829470	H	11.229012	22.700685	10.648791

$Si_{222}Sn_{46}$(17% Sn-shell)				β-Sn_{268}			
H	4.750961	10.698656	20.749502	H	1.496610	10.072108	20.653855
H	0.000000	10.795306	13.472093	H	1.936210	9.128158	17.999687
H	1.600773	6.602453	11.139915	H	14.196930	22.372293	10.302489
H	0.579280	8.839035	11.383007	H	18.684813	21.977324	8.281064
H	6.054186	14.164283	21.980985	H	16.991871	22.623698	10.417514
H	6.322965	1.808003	11.128578	H	4.521427	7.517036	22.061854
H	0.203506	14.019847	16.188703	H	4.959515	5.792294	19.810349
H	3.087255	3.360658	9.006504	H	21.482048	18.627572	10.052741
H	3.447418	14.079316	19.385041	H	19.968046	22.279950	9.952644
H	2.065981	7.679746	15.200132	H	21.323594	22.612601	12.472749
H	1.682985	10.435037	18.175683	H	11.113547	2.437979	20.595724
H	4.626738	2.459572	12.828308	H	25.045739	16.243117	12.692567
H	0.269528	13.351688	10.531787	H	23.128432	20.138552	12.620812
H	4.809484	5.002012	15.095459	H	23.923625	19.243282	15.240141
H	6.008064	3.777202	6.188351	H	17.210817	2.870983	21.064711
H	0.142024	14.609240	11.874509	H	17.455773	3.556320	18.292707
H	5.789572	6.057328	3.416680	H	19.943124	5.508715	18.726000
H	6.152923	19.384244	16.473574	H	22.095086	7.020349	17.715630
H	19.639723	3.588997	13.999566	H	23.280636	8.321462	16.813909
H	8.983861	19.361938	19.312775	H	23.646721	10.267685	14.864437
H	2.017474	15.218858	7.682953	H	24.736172	13.005277	18.064300
H	19.162805	6.446566	16.447776	H	1.287892	19.041430	13.096498
H	1.894537	18.027986	10.230418	H	2.145410	20.026476	15.661962
H	3.151565	19.676249	13.921473	H	4.378837	22.188169	11.001356
H	8.702071	3.583918	3.017238	H	4.587059	23.064623	13.731577
H	9.416261	1.884365	7.574698	H	0.000000	12.890084	17.827280

$Si_{222}Sn_{46}$(17% Sn-shell)				β-Sn_{268}			
H	16.311808	14.812554	22.018062	H	2.171755	16.387302	17.517118
H	10.938722	1.425808	6.466770	H	1.351951	16.102798	20.207983
H	7.417338	1.878769	9.626991	H	3.719489	18.653281	18.074375
H	11.328147	19.828591	22.067578	H	8.038542	23.061303	14.139727
H	3.389164	16.603773	16.538077	H	10.776036	26.023828	12.593348
H	13.286969	17.795761	22.649980	H	10.196085	24.769961	15.128217
H	15.082816	2.451004	7.492216	H	3.791452	13.121715	20.716989
H	3.454665	6.314620	6.226294	H	17.204478	23.094759	14.090414
H	17.988501	1.939153	9.908291	H	8.314614	6.628881	23.118757
H	1.841143	13.196390	4.679377	H	20.605577	23.078550	14.442237
H	16.322450	6.465347	19.319510	H	20.951605	22.250529	17.146263
H	17.406821	13.372674	22.407502	H	10.892778	7.797098	25.868247
H	13.571752	11.085661	24.184377	H	10.540966	6.027015	23.666723
H	1.608673	7.717655	9.696045	H	14.206628	5.920602	22.577797
H	12.471488	15.669783	22.936221	H	23.759093	15.843246	15.002656
H	3.008938	8.745955	3.302608	H	23.198944	17.757761	16.875853
H	15.560548	12.985060	23.207960	H	17.435809	7.537961	25.936201
H	6.163137	16.567295	19.293479	H	17.502852	6.451404	23.303107
H	8.943285	16.686375	22.021097	H	19.982590	5.991337	22.979464
H	0.889440	11.213218	6.560412	H	21.241952	6.883105	20.554668
H	13.263630	0.204114	10.497413	H	21.080674	10.279113	23.530131
H	11.325008	1.074937	8.540470	H	22.962034	9.204529	21.482061
H	10.834844	13.936093	23.765045	H	24.423542	10.158741	19.234197
H	16.401632	0.254954	14.004918	H	23.180091	13.015621	22.952281
H	14.395525	0.000000	11.973284	H	3.984963	22.635234	15.775284
H	16.563960	3.388945	16.743573	H	5.356613	22.179130	18.277823

$Si_{222}Sn_{46}$(17% Sn-shell)				β-Sn_{268}			
H	24.079769	13.520098	10.921539	H	4.911292	20.216204	19.823177
				H	4.607681	18.457905	22.080346
				H	8.171511	22.794280	17.833953
				H	6.711793	22.083776	20.211146
				H	11.097510	22.422155	17.975352
				H	15.017034	24.831088	13.115141
				H	14.417027	26.038276	15.669626
				H	5.199933	9.241002	24.291224
				H	3.974353	10.214552	21.906010
				H	4.714666	12.978856	25.765933
				H	14.072213	22.739909	17.676293
				H	17.212074	24.575155	16.379616
				H	7.679882	10.339474	27.113952
				H	8.665808	9.233328	24.694681
				H	22.105263	19.055228	17.889752
				H	19.836857	20.523932	18.695634
				H	14.123485	4.856256	25.120633
				H	15.110197	9.494796	26.101022
				H	18.185665	13.020401	25.298235
				H	21.060556	15.737010	23.527801
				H	24.575114	15.894469	19.431350
				H	22.784344	16.845170	21.413039
				H	4.005146	15.774925	21.893235
				H	5.211049	16.751731	24.287196
				H	8.315934	19.419249	23.067094
				H	11.115790	23.567019	20.630692

$Si_{222}Sn_{46}$(17% Sn-shell)				β-Sn_{268}			
				H	8.647615	16.720609	24.703149
				H	7.670802	15.614477	27.128659
				H	10.532931	19.944531	23.602656
				H	10.822262	18.227500	25.852600
				H	14.163532	20.118928	22.595506
				H	17.388054	22.476038	18.271306
				H	17.216389	23.136312	21.070010
				H	11.701699	12.095938	27.708419
				H	11.580226	9.250950	28.256957
				H	15.113027	16.500178	26.096662
				H	17.487611	19.576982	23.277684
				H	17.447933	18.506616	25.916589
				H	21.224641	19.157511	20.533179
				H	19.982009	20.035842	22.962624
				H	11.570480	16.738695	28.253452
				H	11.704632	13.889888	27.710129
				H	14.063866	21.144592	25.179335

References

S1 Brus, L. Electronic wave functions in semiconductor clusters: Experiment and theory. *J. Phys. Chem.* **1986**, *90*, 2555–2560.

S2 Dexter, R. N.; Lax, B.; Kip, A. F.; Dresselhaus, G. Effective Masses of Electrons in Silicon. *Phys. Rev.* **1954**, *96*, 222–223.

S3 Afsar, M. N.; Button, K. J. Precise Millimeter-Wave Measurements of Complex Refractive Index, Complex Dielectric Permittivity and Loss Tangent of GaAs, Si, SiO2, Al2O3, BeO, Macor, and Glass. *IEEE Trans. Microw. Theory Techn.* **1983**, *31*, 217–223.

S4 Ng, C. Y.; Chen, T. P.; Ding, L.; Liu, Y.; Tse, M. S.; Fung, S.; Dong, Z. L. Static dielectric constant of isolated silicon nanocrystals embedded in a SiO2 thin film. *Appl. Phys. Lett.* **2006**, *88*, 063103.

S5 Bragg, P. W. H.; Bragg, W. L. The Reflection of X-Rays by Crystals. *Proc. R. Soc. A* **1913**, *17*, 428–438.

S6 Na, S.-H.; Park, C.-H. First-principles Study of Structural Phase Transition in Sn. *Journal of the Korean Physical Society* **2010**, *56*, 494–497.

S7 Haq, A. U.; Buerkle, M.; Askari, S.; Rocks, C.; Ni, C.; Švrček, V.; Maguire, P.; Irvine, J. T. S.; Mariotti, D. Controlling the Energy-Level Alignment of Silicon Carbide Nanocrystals by Combining Surface Chemistry with Quantum Confinement. *The Journal of Physical Chemistry Letters* **2020**, *11*, 1721–1728, PMID: 32040322.
